# Protective effects of 4-HBd on blood–brain barrier integrity in MCAO/R model rats based on brain pharmacokinetic characteristics

**DOI:** 10.3389/fphar.2025.1528839

**Published:** 2025-04-08

**Authors:** Jin Feng, Qian Yang, Ming Chen, Yan Wang, Dan Luo, Dongxiong Hu, Jianjun Cheng, Xuelan Song, Xiaonan Zhou, Qingting Meng, Qing Lin, Fangyan He

**Affiliations:** ^1^ Department of Pharmacology, School of Chinese Materia Medica, Yunnan University of Chinese Medicine, Kunming, China; ^2^ School of Basic Medicine, Yunnan University of Chinese Medicine, Kunming, China

**Keywords:** 4-hydroxybenzaldehyde, blood–brain barrier, tight junction, brain pharmacokinetics, microdialysis, single-cell biochemical analyzer

## Abstract

**Objectives:**

This study explored the brain-targeting properties and mechanisms of 4-hydroxybenzaldehyde (4-HBd), the primary active component of *Gastrodia elata*, in mitigating ischemic stroke (IS)-induced injury by preserving blood–brain barrier (BBB) integrity, based on brain pharmacokinetic characteristics.

**Methods:**

The anti-IS effects of the *G. elata* extract were assessed using a rat middle cerebral artery occlusion/reperfusion (MCAO/R) model, leading to the identification of 4-HBd as the principal active ingredient. BBB protection was evaluated through neurological scoring, Evans Blue (EB) extravasation, cerebral infarct volume, and ultrastructural integrity. Oxidative stress markers, including superoxide dismutase (SOD), malondialdehyde (MDA), nitric oxide (NO), and inducible nitric oxide synthase (iNOS), were quantified in ischemic brain tissue via biochemical assays. The expression levels of tight junction (TJ) proteins claudin-5 and occludin, as well as matrix metalloproteinase MMP-2/9 and aquaporin-4 (AQP-4), were analyzed by Western blotting. Microdialysis, combined with liquid chromatography-tandem mass spectrometry (LC-MS/MS), was employed to determine the temporal distribution of 4-HBd in the brains of both normal and MCAO/R model rats. The ability of 4-HBd to scavenge intracellular reactive oxygen species (ROS) in brain endothelial cells (bEnd.3) was evaluated using a single-cell biochemical analyzer.

**Results:**

*G. elata* ethanol extract exhibited significant anti-IS effects. When compared with the model group, 4-HBd treatment markedly alleviated BBB disruption and neurological deficits, suppressed oxidative stress in ischemic brain tissue, reduced MDA and NO levels, and enhanced SOD activity. The expressions of claudin-5, occludin, MMP-2/9, and AQP-4 were significantly upregulated in the 4-HBd group relative to the model group. Additionally, 4-HBd selectively eliminated nuclear-derived ROS. Pharmacokinetic analysis demonstrated that 4-HBd preferentially accumulated in the striatum and cortex of both normal and MCAO/R model rats. Under ischemic conditions, 4-HBd exhibited accelerated cortical penetration, increased exposure, and prolonged retention.

**Conclusion:**

These findings indicate that 4-HBd exerts a pronounced brain-targeting effect and preserves BBB integrity via the RNS/ROS-MMP-TJ signaling pathway, highlighting its potential as a therapeutic agent for IS.

## 1 Introduction

Stroke is the second leading cause of death and the third leading cause of disability worldwide, with ischemic stroke (IS) accounting for the highest incidence among cerebrovascular diseases (The et al., 2023; Peng et al., 2022; B, 2022). IS is characterized by ischemia-induced neuronal death and neurological dysfunction resulting from the interruption of cerebral blood supply (Guo et al., 2023). Following stroke onset, a series of temporally distinct pathological events occur in the ischemic region, involving multiple pathological mechanisms. In the acute phase, oxidative stress triggers an ischemic cascade, leading to blood–brain barrier (BBB) disruption. The subacute phase is marked by sustained apoptosis and inflammatory responses, while the chronic phase involves activation of endogenous repair mechanisms.

Current pharmacological approaches to IS primarily focus on thrombolysis and neuroprotection (Schuster et al., 2023; Liu and Jia, 2023). Clinical studies have demonstrated that tissue plasminogen activator (tPA) effectively dissolves thrombi, restoring cerebral perfusion and rescuing neurons when administered within 4.5 h of stroke onset (Moran and Rubin, 2022; Havenon et al., 2023). However, due to the stringent therapeutic time window, only 3.4%–5.2% of patients are eligible for tPA treatment (Duan et al., 2023). Furthermore, despite extensive research, no independent neuroprotective agent has demonstrated conclusive clinical efficacy (Buchan and Pelz, 2022). Given these limitations, the development of novel therapeutic strategies and pharmacological interventions remains a critical priority.

**FIGURE 1:**
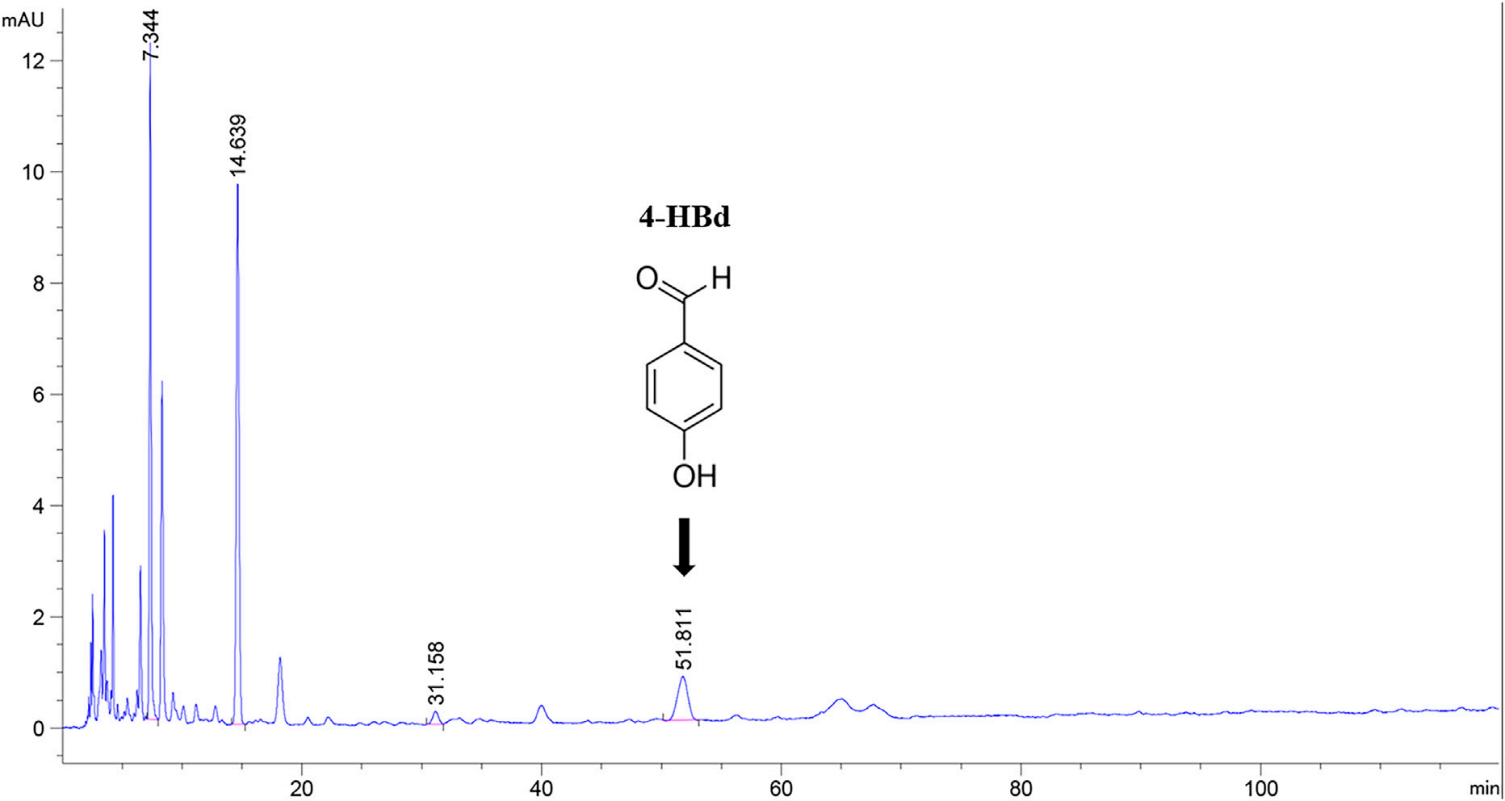
High-performance liquid chromatography of component GE2-1 from *Gastrodia elata*. The peak shape marked with position 4-HBd in the figure has a peak time of 51.811 min.

*Gastrodia elata* Bl. is a well-recognized traditional Chinese medicinal herb widely used for its therapeutic effects in managing convulsions, dizziness, headaches, numbness, and paralysis (Zhao et al., 2023). Due to its well-established safety profile and health benefits, *G. elata* was officially designated as both a food and medicinal substance by China’s National Health Commission and the State Administration of Market Supervision in November 2019. A pilot program for its commercial production and use was subsequently launched in Yunnan, Guizhou, and Sichuan provinces (Guo et al., 2022). Beyond its general health benefits, extensive research over the past 5 decades has focused on the bioactive components and pharmacological efficacy of *G. elata*, particularly following the discovery of its symbiotic relationship with specific microorganisms. However, most investigations have concentrated on gastrodin (GAS), primarily exploring its sedative and hypnotic effects (Long et al., 2021; Dai et al., 2023). Notably, traditional applications of *G. elata* include treatment of headaches, dizziness, seizures, muscle spasms, and impaired speech—symptoms that closely resemble speech impairment, cognitive dysfunction, and motor deficits in IS patients (Ning et al., 2022). This study systematically evaluated the pharmacodynamic properties and bioactive constituents of *G. elata* in the context of IS, demonstrating that its ethanol extract effectively mitigated cerebral ischemia–reperfusion injury (CIRI) in middle cerebral artery occlusion/reperfusion (MCAO/R) model rats (Duan et al., 2015; Xia et al., 2021; Sun et al., 2022). Optimization of the extraction process led to the isolation of an active fraction, GE2-1, which exhibited significant anti-IS activity. Further compositional analysis and efficacy verification identified 4-hydroxybenzaldehyde (4-HBd) as a major bioactive component with strong anti-IS properties, in addition to GAS, which has been more extensively studied (Figure 1).

The BBB relies on the structural integrity of tight junction (TJ) proteins, such as claudin-5 and occludin, to regulate permeability and maintain selective transport. During the pathological process of IS, excessive accumulation of reactive oxygen species (ROS) and nitric oxide (NO), coupled with dysregulated expression of matrix metalloproteinase (MMP) and aquaporin-4 (AQP-4), disrupts TJ proteins, leading to BBB dysfunction. This study investigated the anti-IS effects of 4-HBd in BBB preservation using a rat MCAO/R model. The RNS/ROS-MMPs-TJ signaling pathway was examined as a mechanistic framework to elucidate the anti-IS properties of 4-HBd. Additionally, pharmacokinetic profiling was conducted to characterize its distribution in the brain, while its ROS-scavenging capacity at the single-cell level was assessed. These analyses aimed to clarify the molecular basis of its anti-IS activity and establish the neuroprotective potential of *G. elata* from a brain pharmacokinetics perspective.

## 2 Methods

### 2.1 Isolation and characterization of active constituents in the GE2-1 fraction of *Gastrodia elata*

The GE2-1 fraction was extracted from *G. elata* rhizomes collected in Zhaotong, Yunnan, using 70% ethanol reflux extraction and D101 macroporous resin purification, yielding an extraction efficiency of 3.6%. Reference standards containing GAS (Aladdin) and 4-hydroxybenzyl alcohol (4-HA, J&K Scientific) were dissolved in 3% acetonitrile to final concentrations of 0.162 mg/mL and 0.197 mg/mL, respectively. Parallel preparations of 4-HBd and 3,4-dihydroxybenzaldehyde (3,4-DD) were formulated at 0.1 mg/mL using the same solvent. All solutions were filtered through organic membranes and analyzed using an Agilent 1260 high-performance liquid chromatography (HPLC) system with gradient dilution. For sample analysis, 1.02 mg of GE2-1 was reconstituted in 3% acetonitrile and diluted to a final volume of 1 mL prior to HPLC quantification.

### 2.2 Animals and cells

Adult male Sprague–Dawley rats (250–300 g) were housed under controlled environmental conditions (25°C ± 1°C, 65%–70% humidity, 12-h light/dark cycle) with *ad libitum* access to food and water. All experimental protocols complied with the NIH Guidelines for Animal Care and were approved by the Animal Ethics Committee of Yunnan University of Traditional Chinese Medicine (Approval No. R-062016003), ensuring minimal animal distress.

#### 2.2.1 Model replication

The MCAO/R model was established following our previously published protocol (He et al., 2013). Following 12 h of preoperative fasting, rats were anesthetized with 5% isoflurane anesthesia (RWD) and subjected to right common carotid artery (CCA) isolation. After ligating the CCA and external carotid artery (ECA), a silicone-coated filament was advanced 18 ± 2 mm into the internal carotid artery (ICA) to induce ischemia. Reperfusion commenced after 2 h of occlusion, with core temperature maintained at 37.0°C ± 0.5 °C throughout. Sham controls underwent identical procedures except for filament insertion. Successful occlusion was verified by laser speckle imaging (LSI), defined as a >50% reduction in cerebral blood flow (CBF).

#### 2.2.2 bEnd.3 replication of hydrogen peroxide (H_2_O_2_) model

Murine brain endothelial (bEnd.3) cells were seeded onto 35-mm glass plates at optimal density (1 mL/plate) and cultured under standard CO_2_ conditions. Following adhesion and morphological stabilization, the cells were washed three times with phosphate-buffered saline (PBS) to remove the residual culture medium. Oxidative stress was induced by exposure to 0.5 mM H_2_O_2_ for 4 h in a CO_2_ incubator, establishing the cellular damage model.

### 2.3 Effects of GE2-1 on neural function recovery in a rat IS model

The therapeutic potential of GE2-1 in neural repair following IS was assessed using an MCAO/R rat model. Male Sprague–Dawley rats were randomly assigned to three groups: Sham, Model, and GE2-1 (262.3 mg/kg) (n = 6 per group). Beginning 6 h post-surgery, animals received daily intragastric administration of GE2-1 (*G. elata* ethanol extract) or solvent (0.2% Tween-80 solution) (1 mL/100 g body weight) for 28 days. The CBF was continuously monitored, and neurological function was evaluated weekly using various behavioral tests, including the Longa score, modified neurological severity score, beam walking, rope climbing, gait analysis, and corner test (Supplementary Material S1). The cerebral infarct volume was quantified 24 h post-reperfusion using 2,3,5-triphenyltetrazolium chloride (TTC) staining, with corrected infarct volume calculated as follows: volume (mm^3^) = infarct area × 2; corrected volume = volume × (contralateral/ipsilateral ratio); infarction rate (%) = (corrected volume/total bilateral volume) × 100. For histopathological analysis, coronal brain sections (second–fourth slices) were paraffin-embedded, sliced (5 μm), and subjected to hematoxylin and eosin (H&E) and Nissl staining. Nissl body density was quantified in five randomly selected cortical fields per section.

### 2.4 Molecular docking of 4-HBd with proteins involved in BBB protection and neural repair

Molecular docking analysis was conducted to predict the interactions between 4-HBd and target proteins associated with BBB integrity and neuronal recovery. The two-dimensional (2D) structure of 4-HBd was retrieved from PubChem (https://pubchem.ncbi.nlm.nih.gov/; CID: [126], mol2 format), while 3D protein structures were sourced from UniProt and Protein Data Bank (PDB). Protein preparations included solvent/ligand removal using PyMOL and structural optimization (hydrogen addition, charge calculation, and atom typing) using AutoDock Tools v1.5.6, with final structures exported as PDBQT files. Docking simulations were performed using AutoDock Vina v1.1.2 to assess ligand-protein binding affinities, followed by conformational analysis and visualization in Discovery Studio 2020.

### 2.5 Effects of 4-HBd on BBB protection and neural repair in a rat IS model

To evaluate the role of 4-HBd in BBB stabilization and neuroprotection, the MCAO/R model was replicated, and rats in each group (n = 6 per group) were pretreated with 4-HBd or solvent (0.2% Tween-80 solution) for 7 days (10 or 20 mg/kg). Neurological function was evaluated at 2 h post-ischemia, coinciding with peak BBB permeability, using the Longa 5-point scale, followed by Evans Blue (EB) extravasation and cerebral infarct volume measurement (TTC staining with edema correction) after 24 h of reperfusion. For EB quantification, brains were desiccated at 105°C for 24 h, and EB content was extracted with formamide (50°C, 24 h; 1 mL/hemisphere). Spectrophotometric detection at 610 nm was performed, and EB accumulation was expressed as μg/g dry weight. Frontal and parietal cortical sections were examined using transmission electron microscopy (JEM-1200EX, 80 kV) after dual perfusion with heparinized saline/4% paraformaldehyde, followed by uranyl acetate–lead citrate staining. Oxidative stress markers, including superoxide dismutase (SOD), malondialdehyde (MDA), nitric oxide (NO), and inducible nitric oxide synthase (iNOS), were quantified in homogenized ischemic tissues (1:10 w/v in PBS, 5 000 *g*, 4°C, 5 min) using commercial kits. Western blotting was performed to detect occludin (1:1 000), claudin-5 (1:400), MMP-2 (1:200), and MMP-9 (1:100). Protein samples were transferred to polyvinylidene fluoride (PVDF) membranes, blocked with 5% skim milk, and incubated with horseradish peroxidase (HRP)-conjugated secondary antibodies (1:1 000), followed by chemiluminescence detection.

### 2.6 Tissue distribution of 4-HBd in rats

An HPLC approach was applied to quantify the biodistribution of 4-HBd (400 mg/kg oral dose) in major organs (heart, liver, spleen, lung, kidney, and brain) of normal and MCAO/R model rats during the equilibrium (4 min) and elimination (1 h) phases. Chromatographic separation was achieved using a ZORBAX SB-C18 column (4.6 × 250 mm, 5 μm) with isocratic elution (acetonitrile: 0.1% acetic acid = 10:90 v/v) at 1.0 mL/min, 25°C, and 278 nm detection. Tissue homogenates (1:3 w/v in saline) were subjected to protein precipitation with methanol, nitrogen drying, and reconstitution in the mobile phase prior to HPLC injection (20 μL). Method validation data are provided in Supplementary Material S2.

### 2.7 Brain targeting and pharmacokinetics of 4-HBd

A multi-tiered analytical strategy combining HPLC spatial mapping and LC-MS/MS temporal profiling was implemented to characterize the cerebral pharmacokinetics of 4-HBd (400 mg/kg oral) in normal and MCAO/R model rats. Initial HPLC screening (ZORBAX SB-C18, 4.6 × 250 mm, 5 μm) with isocratic elution (acetonitrile: 0.1% acetic acid = 15:85 v/v) at 1.0 mL/min identified the striatum as the principal site of 4-HBd accumulation (4 min post-administration). Targeted microdialysis was conducted using MD-2200 probes (BASi) stereotaxically implanted into the right striatum (AP: +0.8 mm, ML: +3.0 mm, DV: 3.5 mm) under normothermic anesthesia (37°C ± 0.5°C). Artificial cerebrospinal fluid was perfused over 24 h, with sampling intervals set at follows: 0, 0.25, 0.5, 0.75, 1, 1.25, 1.5, 1.75, 2, 2.5, 4, 6, 8, 10, 12, 14, 16, 18, 20, 22, and 24 h. In normal rats, brain dialysate collection commenced 6 min post-4-HBd gavage, while in MCAO/R model rats, 4-HBd was administered 2 h after ischemia and reperfusion, with brain dialysate collection beginning 6 min post-gavage.

Tissue homogenates (heart, liver, spleen, lung, kidney, and brain substructures) were prepared in saline (1:3 w/v, 13 000 rpm/4°C) and processed via ethyl acetate extraction before reconstitution in the mobile phase for HPLC-UV analysis at 278 nm. Brain dialysates (20 μL) were spiked with protocatechuic acid internal standard (2 μL, 2 000 ng/mL) before LC-MS/MS quantification using a Luna C18 column (150 × 2.0 mm, 3 μm). Separation was achieved under a methanol/0.1% formic acid gradient (35°C) and negative electrospray ionization (ESI) detection (spray voltage: −450 V; transitions: 4-HBd m/z 121→92, PCA m/z 153→109). Calibration curves (1–100 ng/mL) and quality control samples were generated through serial dilution of methanol stock (2 mg/mL), with validation confirming the linearity (r^2^ > 0.998) and precision (relative standard deviation (RSD) < 15%). Pharmacokinetic parameters were derived from time–concentration profiles (0–24 h) using Phoenix WinNonlin.

### 2.8 Targeted ROS scavenging of 4-HBd in bEnd.3 cells

To evaluate the ROS-scavenging capacity of 4-HBd in cerebrovascular endothelial cells (bEnd.3), a single-cell analytical approach was employed using an H_2_O_2_-induced oxidative stress model. Cells were categorized into three experimental groups: untreated controls, H_2_O_2_-exposed models, and 4-HBd treatment groups (single or multiple administrations). Following 24 h of pretreatment with 4-HBd (3.125–50 μg/mL), oxidative stress was induced through H_2_O_2_ exposure (concentration optimized based on preliminary studies). Subcellular ROS levels were quantified using 2′,7′-dichlorofluorescein diacetate (DCFH-DA) fluorescence imaging with a single-cell analyzer. Cellular compartments (membrane, cytoplasm, and nucleus) were precisely targeted using XYZ-axis positioning under 490 nm excitation (50% intensity). Detection parameters included a 1 000 ms gating time, 60 s acquisition window, and 10-ms stabilization period. Pharmacological interventions consisted of microinjections (50 μL) of 4-HBd-containing medium, administered either as a single bolus or sequentially (six injections at 5-min intervals), with parallel recording of photon emission changes for 33 min.

### 2.9 Statistical analysis

Statistical analyses were performed using IBM SPSS Statistics v26 and GraphPad Prism v9. Data normality was assessed using the Shapiro–Wilk test, with the non-parametric Mann–Whitney U test applied for non-normal distributions. Normally distributed data were subjected to homogeneity of variance assessment (Levene’s test) before one-way analysis of variance (ANOVA), followed by least significant difference (LSD) *post hoc* analysis (for homogeneous variance) or Tamhane’s T2 test (for heterogeneous variance). Independent sample *t*-tests were used to compare two-group datasets. Results are presented as mean ± standard deviation (SD), with statistical significance defined as *P* < 0.05.

## 3 Results

### 3.1 Quantification of active components in the *Gastrodia elata* GE2-1 extract

The concentration of bioactive compounds in the GE2-1 fraction was determined at 270 nm. The combined content of active components (GAS + 4-HBd +3,4-DD) was 18.29%, with 4-HBd identified as a key indicative component of the extract (Supplementary Material S3).

### 3.2 Effects of administration timing of GE2-1 on MCAO/R rats

**FIGURE 2:**
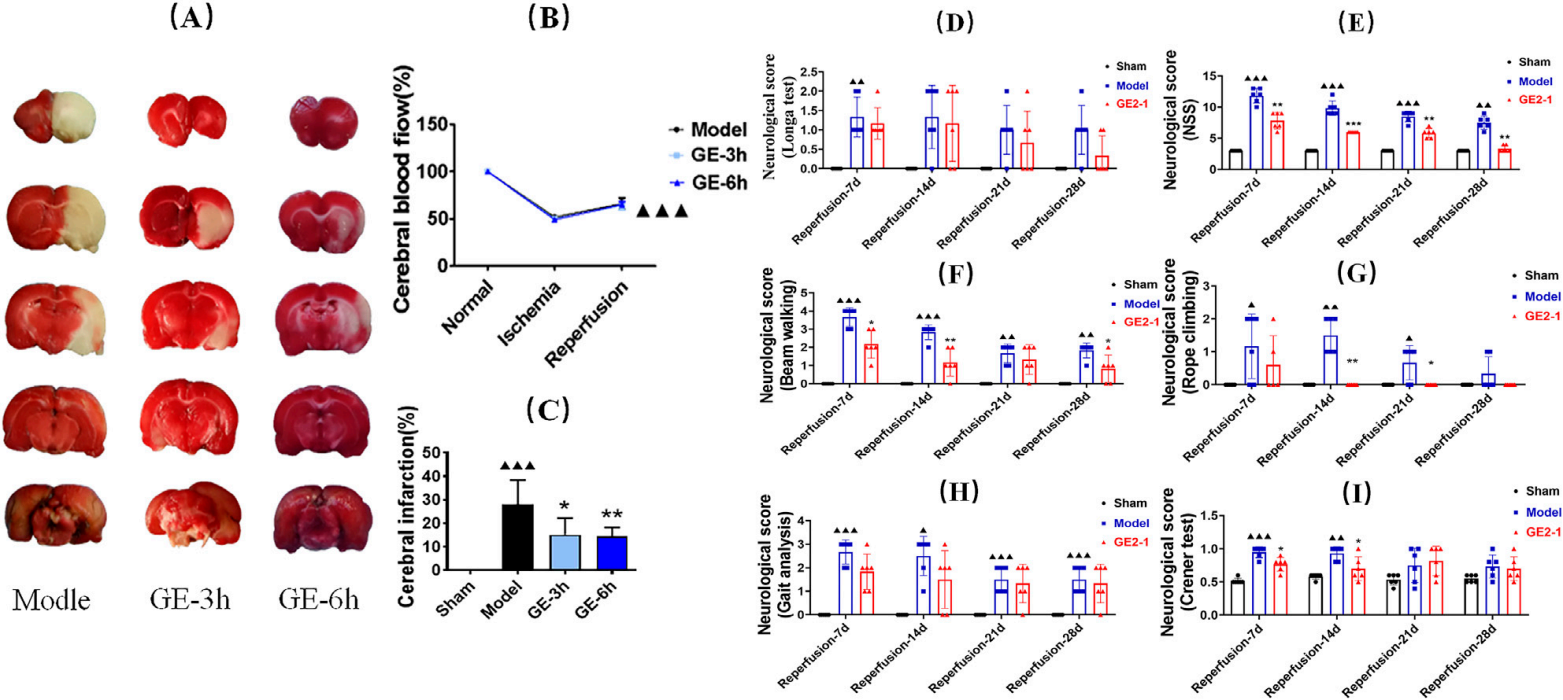
Effect of GE2-1 on brain injury and neural function repair in MCAO/R model rats. **(A)** Effect of GE2-1 intervention at different time points on the cerebral infarction area in MCAO/R model rats, as shown in the statistical results **(C)**; **(B)** Changes in cerebral blood flow before and after the establishment of the MCAO/R model in rats; **(D–I)** effects of GE2-1 on long-term motor function in MCAO/R model rats were tested using **(D)** Longa 5-point, **(E)** NSS score, **(F)** beam walking experiment, **(G)** rope climbing, **(H)** gait analysis, and **(I)** corner test. ^▲▲▲^ *P* < 0.001 vs. preoperative; ^▲▲▲^ *P* < 0.001, ^▲▲^ *P* < 0.005, and ^▲^ *P* < 0.05 vs. Sham group; ^***^ *P* < 0.001, ^**^ *P* < 0.005, and ^*^ *P* < 0.05 vs. MCAO/R group.

Results showed that CBF significantly decreased in the Model, GE-3h, and GE-6h groups within 5 min of ischemia induction compared to baseline levels. Reperfusion restored CBF to over 50% of pre-occlusion levels. Compared with the Model group, rats in the GE-3h and GE-6h groups exhibited a significant reduction in cerebral infarction volume (Figure 2).

### 3.3 Effects of GE2-1 on neural repair in MCAO/R rats

#### 3.3.1 Nervous function

Neurological deficits were significantly more pronounced in the Model group than in the Sham group at 7 and 14 d post-reperfusion. Motor function, assessed using the turning angle test and rope climbing test, showed a progressive decline at 21 and 28 d post-reperfusion. Compared with the Model group, GE2-1 treatment significantly improved neurological recovery, as evidenced by reductions in neurological severity scores and enhanced performance in functional assessments. Notably, by day 7 post-reperfusion, neurological severity, balance beam walking test, and corner test scores had markedly decreased. By day 14, further improvements were observed, with significant reductions in NSS, balance beam walking test, rope climbing test, and corner test scores. By day 21, the neurological severity score and rope climbing test score showed continued improvement, while by day 28, significant reductions were observed in NSS and balance beam walking test scores, indicating progressive motor recovery (Figure 2).

#### 3.3.2 H&E staining

**FIGURE 3:**
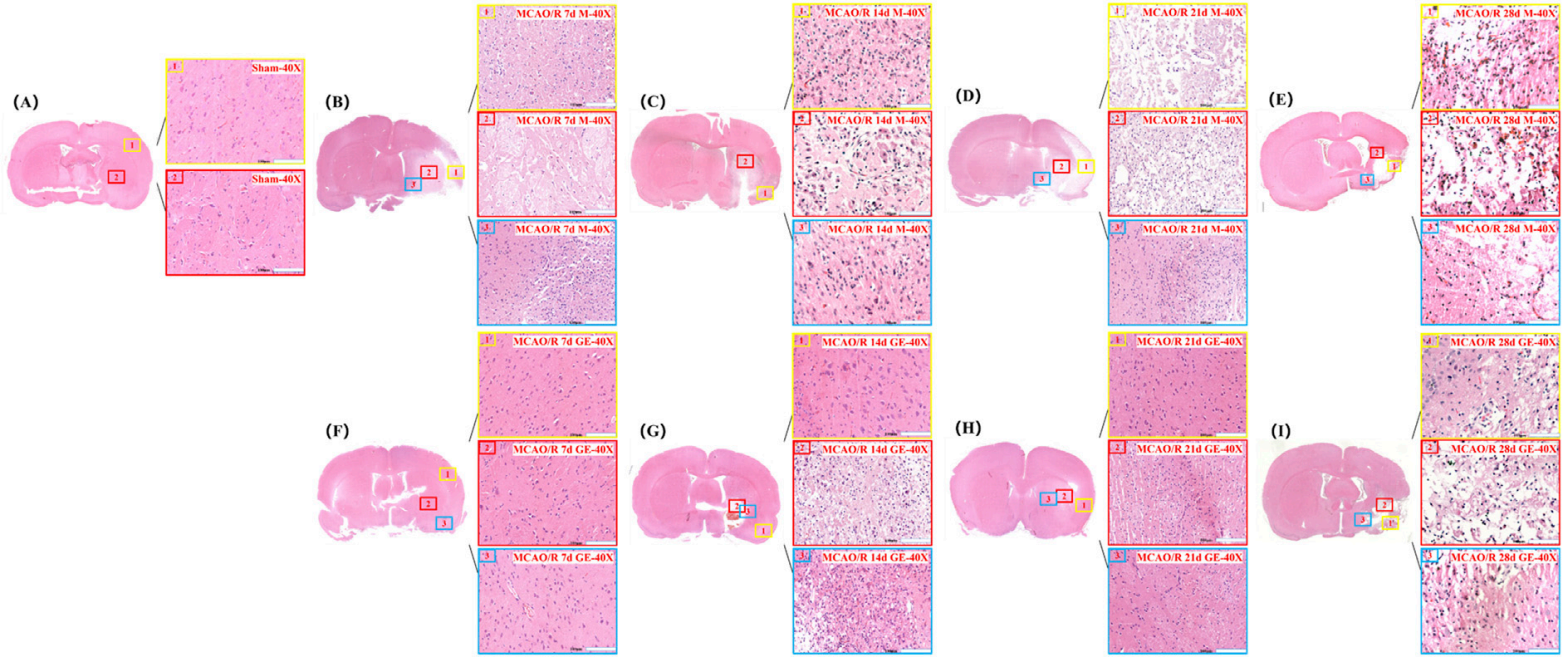
Effect of GE2-1 on HE staining at different time points in rat MCAO/R model. The magnification is ×10 for the eyepiece and ×40 for the objective; **(A)** Sham group; **(B–E)** HE staining was performed on model groups at 7 d, 14 d, 21 d, and 28 d; **(F–I)** HE staining on the GE2-1 group at 7 d, 14 d, 21 d, and 28 d.

In the Sham group, cortical brain tissue exhibited a well-organized structure with intact cells, abundant cytoplasm, clearly defined nucleoli, and a dense, uniform extracellular matrix. In contrast, the Model group displayed extensive ischemic damage on the infarcted side, characterized by multiple regions of focal liquefactive necrosis within the severely affected central area. Cellular density was markedly reduced, with pronounced nuclear pyknosis, hyperchromasia, and nucleolar loss, accompanied by substantial infiltration of inflammatory cells. In the GE2-1 group, neuronal damage was significantly attenuated, with reduced nuclear pyknosis, decreased inflammatory infiltration, and only mild cellular swelling. Overall, brain injury severity was considerably lower compared with the Model group (Figure 3).

#### 3.3.3 Nissl staining

**FIGURE 4:**
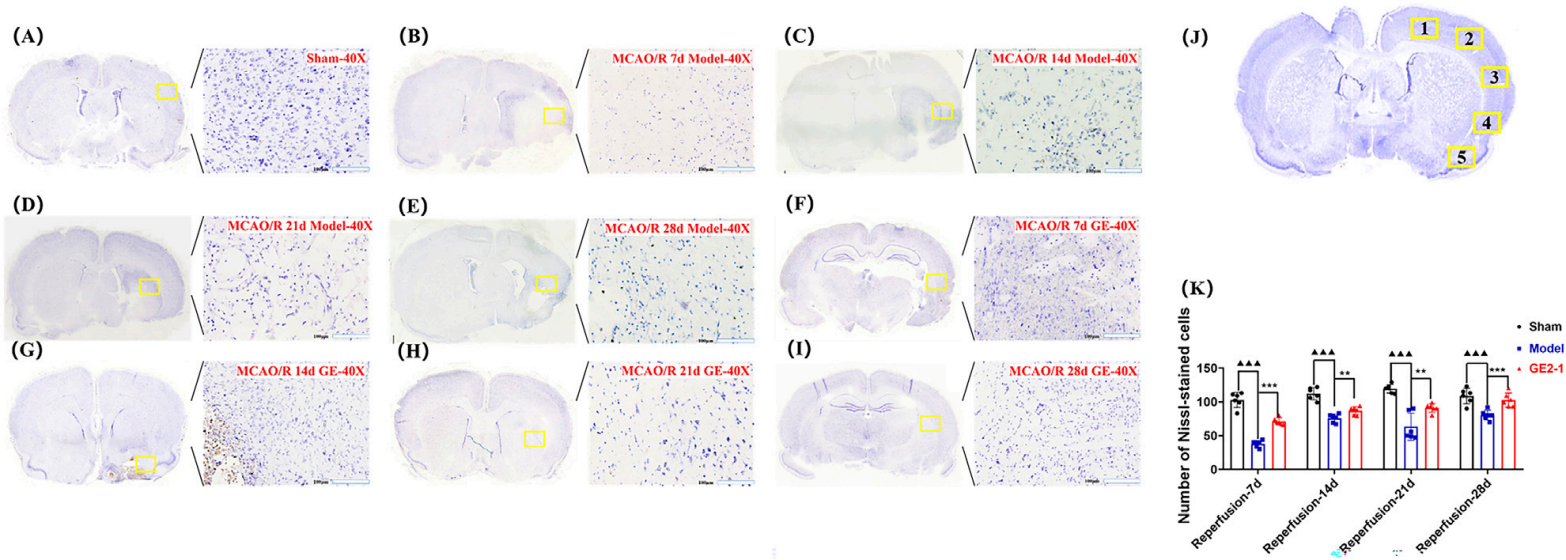
Effect of GE2-1 on Nissl staining at different time points in the rat MCAO/R model. The magnification is ×10 for the eyepiece and ×40 for the objective; **(A)** Sham group; **(B–E)** the model group was stained with Nissl at 7 d, 14 d, 21 d, and 28 d; **(F–I)** GE2-1 group 7 d, 14 d, 21 d, and 28 d Nissl staining. **(J)** Nissl corpuscle counting position. **(K)** Nissl staining of nerve cell count statistics.

The number of Nissl bodies in the ischemic side of the Model group was significantly reduced at 7, 14, 21, and 28 d post-reperfusion compared with the Sham group. However, GE2-1 treatment significantly increased Nissl body density at 7, 14, and 28 d post-reperfusion. In the Sham group, neurons maintained a well-organized arrangement with intact nuclear membranes and nucleoli and cytoplasm densely packed with blue granular Nissl bodies, with no signs of neuronal necrosis. In contrast, the Model group exhibited severe neuronal damage, including pyknosis, hyperchromasia, and triangular or fragmented nuclei, with a substantial loss of Nissl bodies and diminished staining intensity. GE2-1 treatment preserved neuronal integrity, increased Nissl body density, and maintained a relatively uniform cellular structure (Figure 4).

### 3.4 Anti-IS effects of 4-HBd

#### 3.4.1 Molecular docking of 4-HBd

**FIGURE 5:**
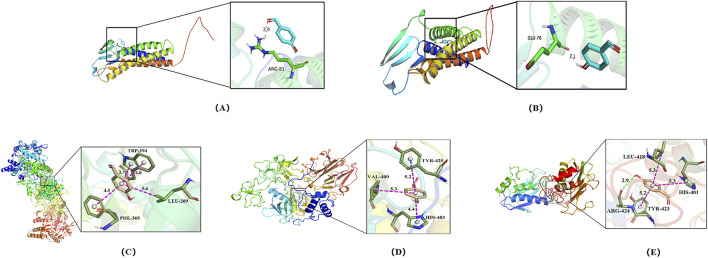
Molecular docking of 4-HBd on BBB protection-related indicators. **(A)** Claudin-5 interaction with 4-HBd; **(B)** occludin interaction with 4-HBd; **(C)** iNOS interaction with 4-HBd; **(D)** MMP-2 interaction with 4-HBd; and **(E)** MMP-9 interaction with 4-HBd.

MMP-2, MMP-9, iNOS, occludin, and claudin-5 were identified as key potential targets of 4-HBd in IS. Therefore, molecular docking experiments were performed to assess the binding affinity between 4-HBd and these target proteins. Docking results showed that 4-HBd exhibited favorable binding interactions with all five selected proteins, with binding energies ranging from −6.2 to −4.9 kcal/mol. Generally, a binding energy <0 kcal/mol suggests a potential interaction, <−5.0 kcal/mol indicates strong binding affinity, and <−7.5 kcal/mol indicates excellent binding ability. Analysis demonstrated that 4-HBd effectively interacted with all five BBB-related proteins, with claudin-5, MMP-2, MMP-9, occludin, and iNOS displaying binding energies greater than −5 kcal/mol, suggesting moderate-to-strong affinity (Figure 5; Table 1).

**TABLE 1:** Components docked with key targets.

Target gene (PDB ID)	Compound	Binding energy, kcal/mol
MMP2 (1CK7)	4-Hydroxybenzaldehyde	−6.2
MMP9 (1GKC)	4-Hydroxybenzaldehyde	−5.3
iNOS (1NSI)	4-Hydroxybenzaldehyde	−6.2
Occludin (1WPA)	4-Hydroxybenzaldehyde	−4.9
Claudin-5 (full chain)	4-Hydroxybenzaldehyde	−5.0

PDB, Protein Data Bank; MMP2/9, matrix metalloproteinase; iNOS, inducible rate-limiting enzymes of NO synthesis; occludin and claudin-5, tight junction proteins.

4-HBd formed a hydrogen bond with the ARG-81 residue on claudin-5 (Figure 5A) and the GLU-76 residue on occludin (Figure 5B). It also established three hydrophobic interactions with TRP-194, PHE-369, and LEU-209 on iNOS (Figure 5C); TYR-425, VAL-400, and HIS-430 on MMP-2 (Figure 5D); and TYR-423, LEU-418, and HIS-410 on MMP-9, along with an additional hydrogen bond at ARG-424 (Figure 5E).

#### 3.4.2 Regulation of BBB ultrastructure following 4-HBd treatment

**FIGURE 6:**
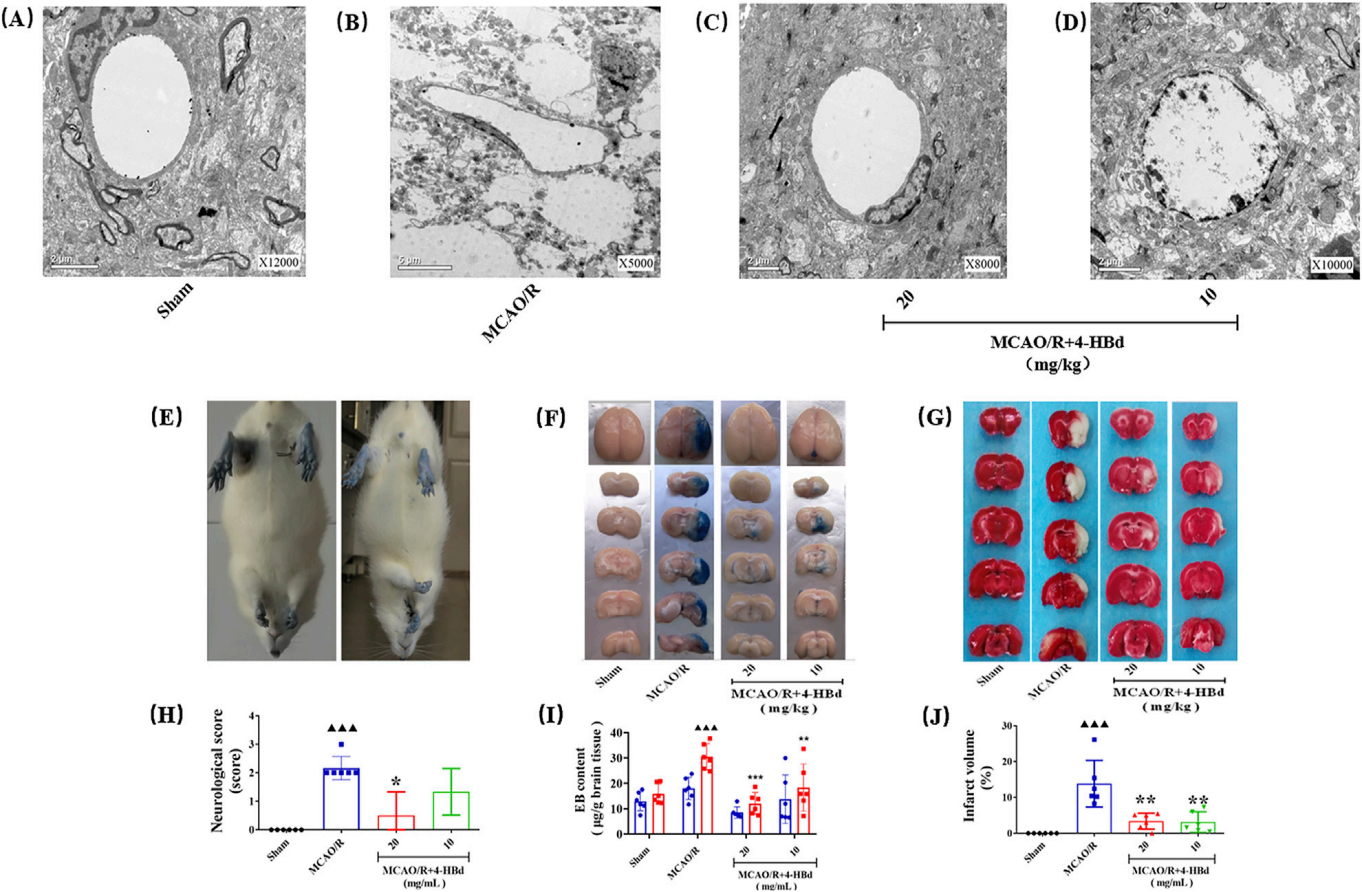
Neurorepair and BBB-protective effect of 4-HBd on MCAO/R model rats. **(A)** Electron microscopy was used to observe the ultrastructural morphology of the BBB in each group of rats; **(B)** neurological morphological changes of rat limbs during tail lifting suspension; **(C)** effect of 4-HBd on the permeability of EB in rat brain tissue; **(D)** effect of 4-HBd on the cerebral infarction area in MCAO/R model rats; and **(E-H)** statistical results are as follows:neurological score **(E)**, Evans blue content **(F, I)**, and cerebral infarction area **(G, J)**.

Ultrastructural changes in the BBB were assessed 24 h after cerebral ischemia–reperfusion (Figure 2A). In the Sham group, the BBB exhibited a well-preserved structure, consisting of an intact basal lamina, endothelial cells, TJs, perivascular astrocytic end-feet, and capillaries. However, in the MCAO/R group, BBB integrity was severely compromised, with microvascular luminal narrowing, compression, and pronounced perivascular edema in astrocytic end-feet. Pretreatment with 4-HBd (10 mg/kg and 20 mg/kg) significantly reduced astrocytic swelling and preserved the structural integrity of endothelial cells, basal lamina, and TJs (Figure 6A).

#### 3.4.3 Effects of 4-HBd on neurological deficits in rats

Compared with the Sham group, MCAO/R model rats exhibited obvious neurological deficits, with significant differences in neurological severity scores. Treatment with 4-HBd at 20 mg/kg significantly improved neurological function, with marked reductions in deficit severity compared with the MCAO/R model rats (Figures 6B,E).

#### 3.4.4 Effects of 4-HBd on EB content in rat brains

EB extravasation was assessed to evaluate BBB permeability. No significant EB exudation was observed in the left hemisphere across all groups, and EB content in the left brain tissue showed no significant differences. However, in the right hemisphere of the MCAO/R model group, extensive EB exudation was detected, with a significant increase in EB content, indicating severe BBB disruption. Compared with the Model group, treatment with 4-HBd (10 mg/kg and 20 mg/kg) significantly reduced EB accumulation in the right ischemic brain tissue, suggesting enhanced BBB integrity (Figures 6C, F).

#### 3.4.5 Effects of 4-HBd on cerebral infarct volume in MCAO/R model rats

Compared with the Sham group, MCAO/R model rats exhibited significant cerebral infarction, with a significant increase in the infarct volume. Treatment with 4-HBd (10 mg/kg and 20 mg/kg) significantly reduced infarct volume compared with the Model group (Figures 6D, G).

#### 3.4.6 Effects of 4-HBd on TJ-related protein expression in ischemic brain tissue

**FIGURE 7:**
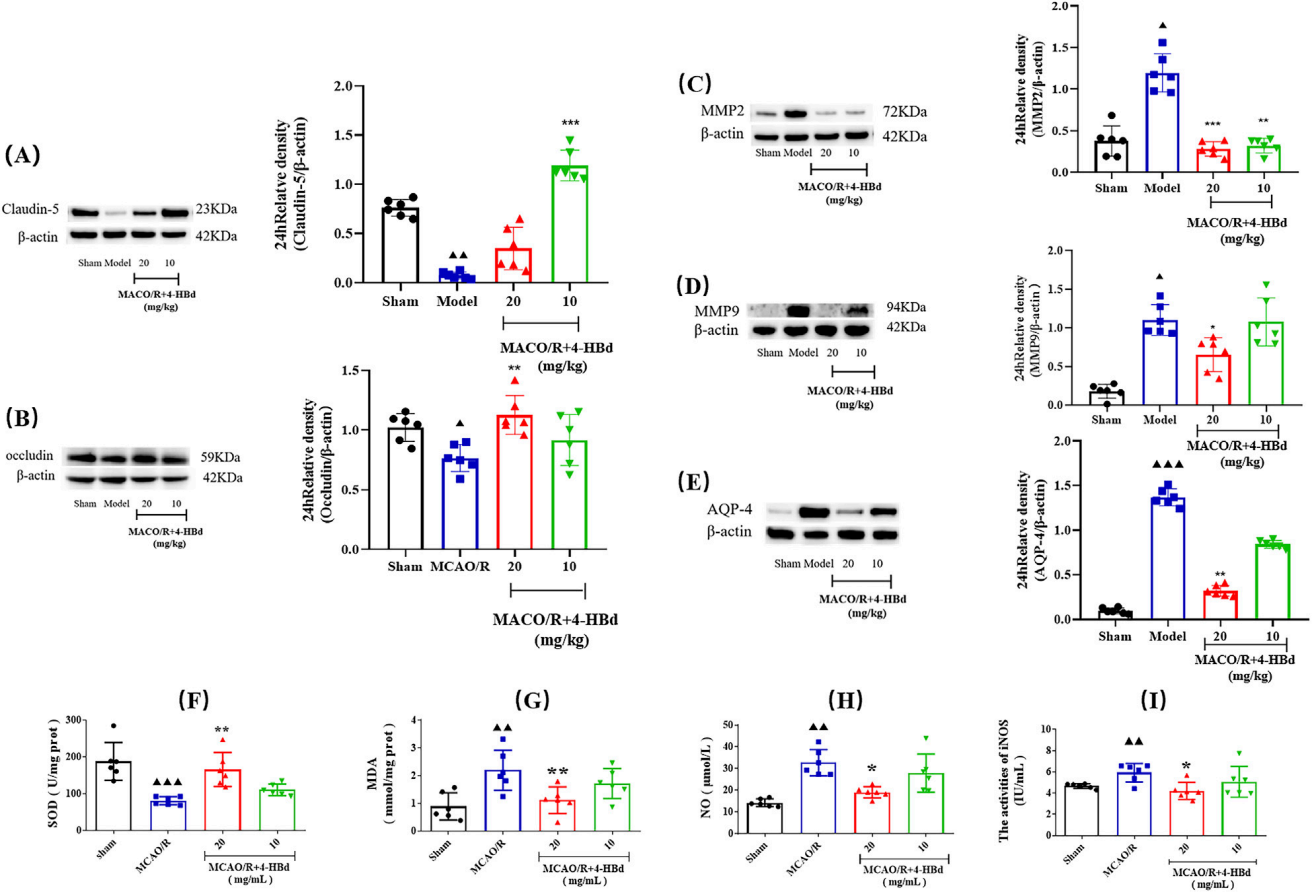
Regulatory effect of 4-HBd on ischemic brain tissue-related targets in MCAO/R rats. **(A)** Claudin-5; **(B)** occludin; **(C)** MMP-2; **(D)** MMP-9; **(E)** AQP-4; **(F)** SOD; **(G)** MDA; **(H)** NO; and **(I)** iNOS. ^▲▲▲^ *P* < 0.001 and ^▲▲^ *P* < 0.005, vs. Sham group; ^**^ *P* < 0.005 and ^*^ *P* < 0.05 vs. MCAO/R group.

Compared with the Sham group, claudin-5 and occludin expressions in ischemic brain tissue were significantly downregulated, while MMP-2/9 and AQP-4 expressions were significantly upregulated in the MCAO/R model group. Treatment with 4-HBd significantly restored claudin-5 and occludin expressions while markedly downregulating the expressions of MMP-2/9 and AQP-4 in ischemic brain tissue, indicating improved BBB integrity (Figures 7A–E).

#### 3.4.7 Antioxidant and reactive nitrogen species modulation by 4-HBd

Assessment of oxidative stress markers demonstrated that, compared with the Sham group, SOD activity was significantly reduced in the ischemic brain tissue of MCAO/R model rats, while MDA, NO, and iNOS were significantly elevated. Treatment with 4-HBd (20 mg/kg) significantly enhanced SOD activity, while significantly reducing MDA, NO, and iNOS in the ischemic brain tissue compared with the Model group (Figures 7F–I).

### 3.5 Tissue distribution of 4-HBd in rats

#### 3.5.1 Method validation for 4-HBd quantification in tissue homogenates

Details of the methodological validation for 4-HBd analysis in rat tissue homogenates are provided in Supplementary Material S2.

#### 3.5.2 Tissue distribution of 4-HBd at equilibrium (4 min) and elimination (1 h) phases

At the equilibrium phase (4 min) following oral administration, the tissue concentration of 4-HBd in normal rats was highest in the brain, followed by the liver, heart, spleen, lung, and kidney. In MCAO/R model rats, the concentration ranking was brain > liver > spleen > heart > kidney > lung. At the elimination phase (1 h), the distribution of 4-HBd in normal rats was the highest in the brain, followed by the lung, kidney, spleen, liver, and heart. In MCAO/R model rats, the concentration order was brain > lung > kidney > spleen > liver > heart (Supplementary Material S4).

### 3.6 Brain targeting analysis of 4-HBd

#### 3.6.1 Method specificity

Chromatographic specificity was evaluated by analyzing 10 μL of the blank dialysate from six rats of different sources. A 20-μL brain dialysate sample containing 100 ng/mL 4-HBd was mixed with 2 μL of PCA internal standard solution (2 000 ng/mL), followed by injection of 10 μL for chromatographic analysis. Similarly, another 20 µL of the dialysate sample was spiked with 2 μL of the PCA internal standard solution (2 000 ng/mL) and subjected to the same analytical procedure. The results demonstrated minimal interference at the retention time of 4-HBd and its internal standard, confirming strong method specificity and well-defined peak resolution (Supplementary Material S5).

#### 3.6.2 Residual rate assessment

The residual rate was evaluated by sequential injection of a high-concentration sample followed by a blank sample. The residual analyte in the blank sample was required to remain below 20% of the lower limit of quantification (LOQ), while the internal standard residue was limited to less than 5%. Results confirmed that residual levels in blank samples following high-concentration sample analysis complied with these acceptance criteria (Supplementary Material S5).

#### 3.6.3 Linearity and standard curve

Serial dilutions of 4-HBd (1 000, 500, 200, 100, 50, 20, and 10 ng/mL) were prepared in 10-μL aliquots, mixed with 90 μL of the rat brain dialysate, and analyzed. The relationship between 4-HBd-to-IS peak area ratio (y-axis) and 4-HBd-to-IS concentration ratio (x-axis) was established, applying a 1/x^2^ weighting factor. The standard curve equation was as follows: Y = 0.0183x + 0.0166 (R^2^ = 0.9964), with a linear range of 1–100 ng/mL, meeting the requirements for quantifying 4-HBd in brain dialysate samples. The lower LOQ was 1 ng/mL, with intra-assay and inter-assay precision of 6.73% and 6.55%, respectively, and an accuracy (relative error, RE%) of −3.1%. These findings confirm that the method is highly sensitive, precise, and reliable for detecting low concentrations of 4-HBd in brain dialysate samples (Supplementary Material S5).

#### 3.6.4 Precision and accuracy

The precision and accuracy of the method were assessed using quality control samples at high, medium, and low concentrations, with six replicates per concentration. Intra-day precision was evaluated by performing six parallel operations within a single day, while inter-day precision was assessed over 3 consecutive days. The RSD% was calculated based on the measured concentrations. For method validation, RSD% for all concentration levels was required to be ≤15%, and accuracy (relative error, RE%) had to remain within ±15%. Results showed that intra-day RSD% for precision did not exceed 3.9%, while inter-day RSD% remained below 8.4%. Furthermore, accuracy (RE%) was within ±8.4%, confirming that the method demonstrated high precision and accuracy, making it suitable for the reliable quantification of 4-HBd in brain dialysate samples (Supplementary Material S5).

#### 3.6.5 Matrix effects

To evaluate potential matrix effects, the blank rat brain dialysate was used to prepare both low- and high-concentration samples, which were then analyzed following established methods. The peak areas of 4-HBd and PCA internal standard were recorded as AS1 (1) and AI (1), respectively. In parallel, equivalent samples were prepared using water instead of the brain dialysate, with peak areas of 4-HBd and PCA recorded as AS1 (2) and AI (2), respectively. The matrix factor for 4-HBd was calculated as AS1 (1)/AS1 (2), while the matrix factor for the internal standard was AI (1)/AI (2). The normalized matrix factor for 4-HBd was then determined by the ratio of these two values. Results indicated that RSD% for low and high concentrations of 4-HBd was 5.471% and 11.230%, respectively, both within the acceptance criterion of ≤15%, confirming that matrix effects did not interfere with the accuracy of 4-HBd quantification (Supplementary Material S5).

#### 3.6.6 Stability assessment

The stability of 4-HBd in dialysate samples was evaluated under various conditions. Low- and high-concentration quality control samples were prepared and stored at room temperature for 8 h, subjected to three freeze–thaw cycles, and stored at −80°C for 12 h and 14 days. Samples were analyzed using the corresponding standard curve to assess the stability. Results demonstrated that the accuracy (RE%) for all tested conditions—including room temperature storage, freeze–thaw cycles, and long-term storage—remained within ±15%, indicating that 4-HBd was stable under these conditions (Supplementary Material S5).

#### 3.6.7 Pharmacokinetics of 4-HBd in the rat brain

**FIGURE 8:**
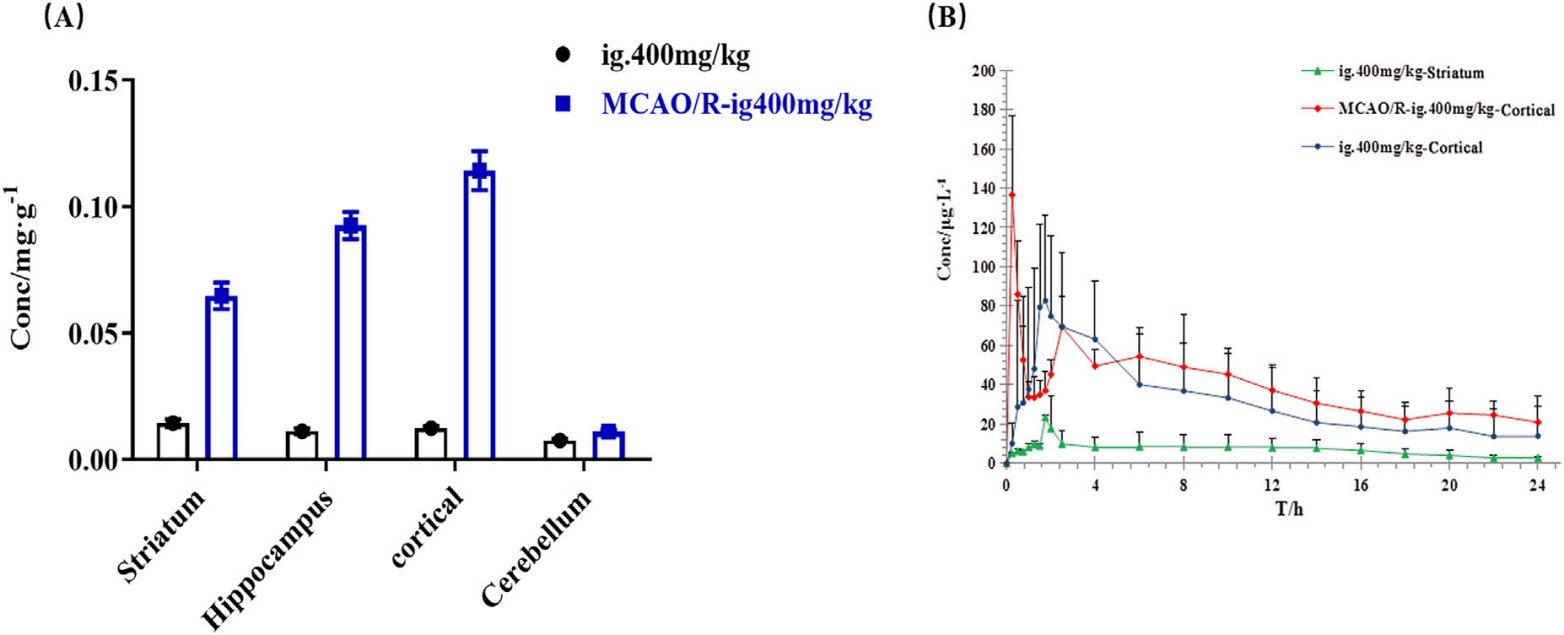
Brain pharmacokinetic characteristics of 4-HBd in the rat brain. **(A)** Distribution of 4-HBd in the brain of rats. **(B)** Brain concentration–time curve of 4-HBd in rats.

Pharmacokinetic analysis of 4-HBd in the striatum and cortex was performed following intragastric administration. In normal rats, the area under the concentration-time curve (AUC_(0–t)_) in the striatum was 648.38 ± 179.62 μg/Lh, with a T_max_ of 1.75 h and t_1/2z_ of 11.29 h. In the cortex, AUC_(0–t)_ was 65.1 ± 22.68 μg/Lh, with a T_max_ of 1.79 h and t_1/2z_ of 5.14 h. These findings indicate that 4-HBd exhibited a higher drug concentration in the striatum compared to the cortex in normal rats. In MCAO/R model rats, AUC_(0–t)_ was 313.77 ± 74.61 μg/Lh, with a T_max_ of 0.25 h and t_1/2z_ of 22.73 h. Notably, after intragastric administration, 4-HBd concentrations in the cortical region of MCAO/R model rats were higher than those observed in normal rats, suggesting altered brain distribution under ischemic conditions (Figure 8; Table 2).

**TABLE 2:** Brain pharmacokinetic parameters of 4-HBd in the rat brain.

Parameter	ig.400 mg/kg-striatum		ig.400 mg/kg-cortical		MCAO/R-ig.400 mg/kg-cortical	
	*x̅* ± *s*	RSD/%	*x̅* ± *s*	RSD/%	*x̅* ± *s*	RSD %
AUC_(0–t)_ μ g · L^−1^ · h	648.38 ± 179.62	27.7	65.1 + 22.68	34.8	313.77 ± 74.61	23.8
AUC 0−∞ / μ g · L^−1^ · h	854.53 ± 293.00	34.3	71.41 ± 27.20	38.1	695.97 ± 347.84	50
MRT_(0–t)_/h	8.13 ± 0.98	12	9.03 ± 2.67	29.6	7.96 ± 2.15	27
VRT_(o–t)_/h^2^	33.55 ± 9.11	27.2	34.06 ± 8.89	26.1	31.10 ± 15.56	50
t_1/2z_/h	11.29 ± 7.27	64.4	5.14 ± 1.67	54.2	22.73 ± 16.41	72.2
T_max_/h	1.75 ± 0	0	1.79 ± 0.10	32.5	0.25 ± 0	0
Vz/F/L · kg^−1^	7846.03 ± 5300.72	67.6	35849.14 ± 5338.93	5.7	17209.72 ± 5859.34	34
CLz/F/L.h^−1^ · kg^−1^	504.18 ± 129.68	25.7	7122.43 ± 5116.23	14.9	681.57 ± 258.04	37.9
C_max_/ μ g · L^−1^	73.89 ± 17.00	23	10.86 ± 4.11	71.8	53.71 ± 5.28	9.8

Notes: Values were presented as mean ± SD (%CV); Abbreviations: AUC_0–t_, area under the curve (AUC) from zero to the last quantifiable concentration point; AUC_0–∞_, AUC from zero to infinity; T_max_, time to C_max_; t_1/2_, the elimination half-life; Cmax, the peak concentration. MRT, average dwell time; VRT, average residence time difference size; VZ/F, apparent volume of distribution; CLzF, clearance rate of oral absorption.

### 3.7 Targeted ROS scavenging by 4-HBd in bEnd.3 cells

#### 3.7.1 Optimization of ROS accumulation target and 4-HBd concentration in the H_2_O_2_-induced bEnd.3 model

**FIGURE 9:**
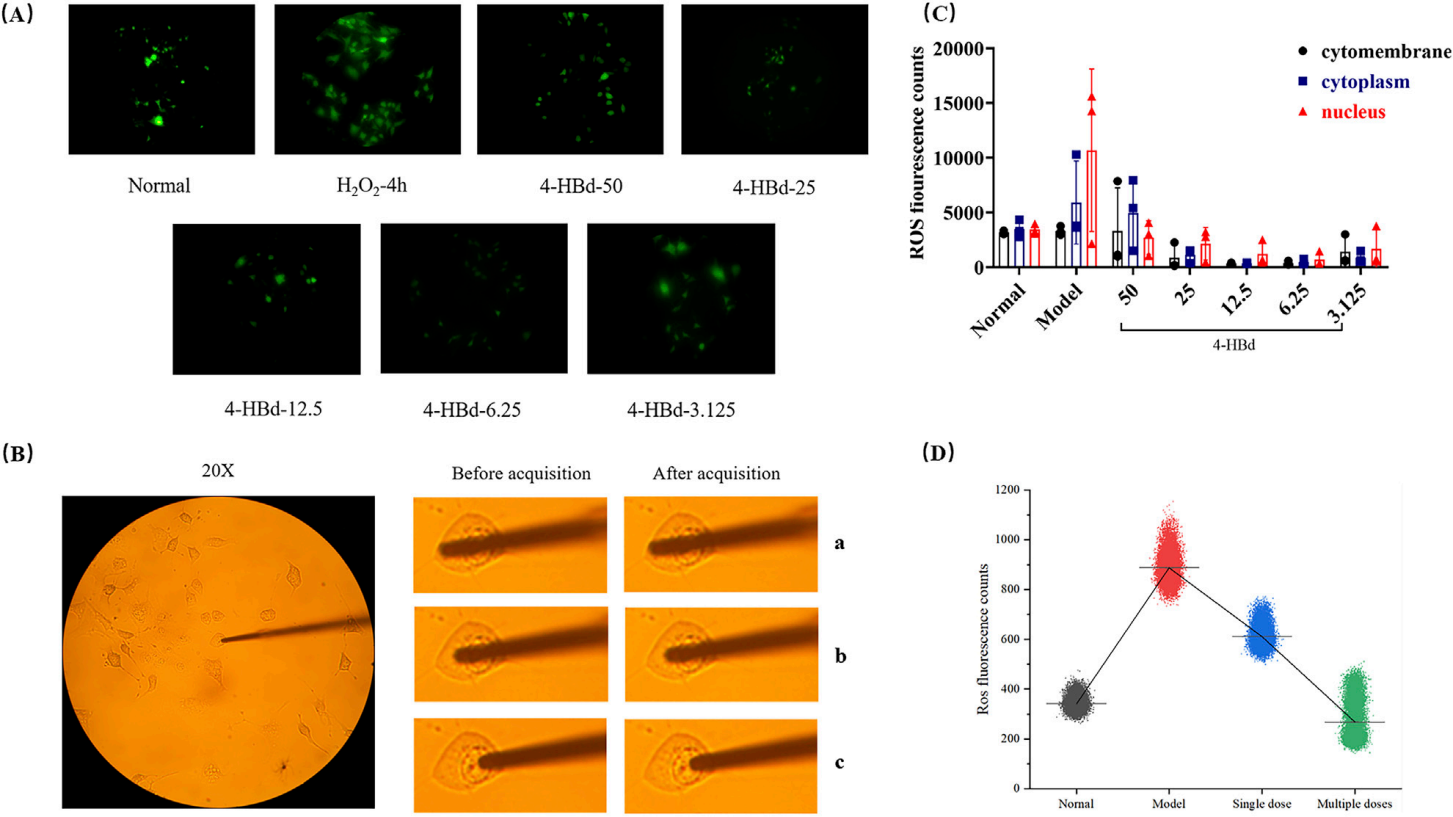
Targeted ROS clearance of 4-HBd at bEnd.3. **(A)** DCFH-DA fluorescence staining of bEnd.3. **(B)** Optical probe positioning diagram of bEnd.3. **(C)** Number of ROS photons at different target sites of bEnd.3 after 4-HBd intervention. **(D)** Effect of 4-HBd on the number of ROS photons in the bEnd.3 H_2_O_2_ model.

ROS accumulation in bEnd.3 cells was assessed using a DCFH-DA fluorescence probe, which exhibited stable green fluorescence across all experimental groups. No significant changes in fluorescence localization were observed before or after image acquisition, ensuring precise targeting and measurement. Compared with the normal group, the H_2_O_2_-induced oxidative stress model exhibited a significant increase in nuclear ROS accumulation. Treatment with 4-HBd effectively reduced ROS photon emission at different cellular target sites, with the most pronounced reduction observed at a concentration of 6.25 ug/mL, which was identified as the optimal 4-HBd dose (Figure 9).

#### 3.7.2 Effects of 4-HBd on nuclear ROS in H_2_O_2_-induced bEnd.3 model

Following 4 h of H_2_O_2_ exposure, ROS accumulation at the nuclear site was significantly elevated at the 33-min collection time point. Single and multiple therapeutic administrations of 4-HBd effectively reduced nuclear ROS levels, with the multi-dose 4-HBd group exhibiting a significant downward trend compared with the single-dose 4-HBd group (Figure 9).

## 4 Discussion

This study demonstrated that 4-HBd exerted significant neuroprotective effects against ischemic brain injury, mitigating both structural damage and neurobehavioral impairments. The therapeutic potential of 4-HBd in cerebral ischemia was evident in its ability to preserve BBB integrity, prevent neuronal degeneration, and improve neurobehavioral deficits. In addition, this study established 4-HBd as a key bioactive component underlying the neuroprotective effects of *G. elata*, highlighting its capacity to attenuate oxidative stress in ischemic brain tissue. Specifically, 4-HBd effectively reduced MDA and NO levels, suppressed iNOS activity, and increased SOD activity, thereby modulating oxidative homeostasis. Furthermore, 4-HBd exerted BBB-protective effects through the RN-MMP-TJ signaling pathway, with its therapeutic action also reflected in its ability to scavenge ROS at the single-cell level.

The BBB is a highly dynamic physiological barrier that maintains cerebral homeostasis by regulating molecular exchange between the systemic circulation and brain tissue. It consists of vascular endothelial cells, TJ proteins, a basement membrane, pericytes, and astrocytic end-feet, all of which contribute to its selective permeability and structural integrity (Fuyuko et al., 2021). Following IS, endothelial swelling and vascular luminal narrowing are among the earliest pathological changes. As the ischemic cascade progresses, further endothelial pyknosis, basement membrane rupture, vascular luminal deformation and stenosis, endothelial apoptosis, and TJ protein degradation occur, leading to compromised BBB integrity, increased permeability, and impaired barrier function (Huang et al., 2020). Electron microscopy results confirmed that 4-HBd reduced BBB damage, preserved vascular luminal integrity, prevented basement membrane degradation, and alleviated astrocytic foot process edema. In addition, 4-HBd significantly improved cerebral infarction in MCAO/R model rats, reduced BBB permeability, and limited EB extravasation, indicating a protective role in BBB structure and function. Multiple factors contribute to BBB disruption during IS, and its underlying molecular mechanisms are complex. The primary drivers of BBB damage and increased permeability include excessive NO accumulation, upregulation of MMPs, overexpression of AQP-4, and degradation of key TJ proteins such as occludin and claudin-5. Additionally, oxidative stress and inflammatory responses exacerbate vascular injury, leading to a multifactorial pathological process (Dai et al., 2018; Danjo et al., 2013). Given this complexity, the identification of bioactive compounds capable of preserving BBB integrity during the early stages of IS is of paramount importance. From a regulatory effector molecule perspective, this study underscores the potential of *G. elata*-derived 4-HBd as a promising candidate for BBB protection in IS.

Excessive NO accumulation is a major contributor to BBB disruption. NO synthesis is regulated by three rate-limiting enzymes: neuronal nitric oxide synthase (nNOS), inducible nitric oxide synthase (iNOS), and endothelial nitric oxide synthase (eNOS). Among these, iNOS continuously produces NO, leading to sustained inflammatory damage, while nNOS-derived NO exhibits neurotoxic properties that aggravate brain injury (Anavi and Tirosh, 2020). Our results showed that 4-HBd protected the BBB by inhibiting iNOS activity, thereby reducing NO synthesis in ischemic brain tissue, mitigating neurovascular damage. MMPs are a family of zinc-dependent proteases that degrade extracellular matrix components and the basement membrane, playing a critical role in BBB integrity. MMP-2 mediates early-stage, reversible BBB damage, leading to TJ loosening, while MMP-9 is involved in late-stage, irreversible BBB damage, contributing to TJ protein degradation and increased permeability (Qiao et al., 2022). Our findings indicated that 4-HBd preserved BBB integrity by downregulating MMP-2 and MMP-9 expressions in ischemic brain tissue, thereby preventing extracellular matrix degradation and excessive permeability.

Tight junctions are fundamental to BBB structural integrity and permeability regulation. BBB function is directly influenced by TJ opening and closing, which is tightly linked to the expression, degradation, and redistribution of key TJ proteins (Yang and Torbey, 2020). The primary transmembrane proteins of TJs, claudin-5 and occludin, regulate paracellular transport of water-soluble molecules and ions across the BBB (Berndt et al., 2019). Our study demonstrated that 4-HBd administration enhanced BBB stability by upregulating the expressions of TJ-related proteins, including occludin and claudin-5, thereby reducing pathological permeability.

AQP-4, the most widely distributed aquaporin in the central nervous system, is localized on brain capillary endothelial cells and astrocyte foot processes, forming a crucial interface between the BBB and the surrounding neurovascular environment. AQP-4 is integral to BBB opening, brain edema formation, and fluid clearance (Ji et al., 2015; Kuruca et al., 2017). Elevated AQP-4 expression is associated with increased brain edema severity, exacerbating ischemic damage. Our findings demonstrated that 4-HBd intervention reduced AQP-4 expression levels, effectively inhibiting edema progression and contributing to neurovascular protection.

Based on animal experiments, the tissue distribution profile of 4-HBd was examined following administration, revealing its strong brain-targeting properties. During both the elimination and equilibrium phases, 4-HBd exhibited the highest concentration in brain tissue in both normal and MCAO/R model rats. Pharmacokinetic analysis further demonstrated that the brain regions with the highest 4-HBd exposure and longest retention were in the striatum in normal rats and the cortex in MCAO/R model rats. The striatum plays a crucial role in voluntary movement stability, muscle tension regulation, and limb posture control, while the cortex functions as the primary center for motor regulation, cognition, language, memory, and spatial perception. Notably, CIRI significantly altered the distribution characteristics of 4-HBd, leading to increased 4-HBd concentrations across all brain regions. This phenomenon may be attributed not only to increased BBB permeability under pathological conditions but also to potential target interactions of 4-HBd within ischemic brain regions. To further explore the targeted effects of 4-HBd at the cellular level, this study examined brain microvascular endothelial cells (bEnd.3) exposed to oxidative stress induced by H_2_O_2_. Results confirmed that 4-HBd exhibited potent ROS-scavenging activity, with a marked ability to selectively eliminate nuclear ROS in bEnd.3 cells.

This study investigated the protective effects and molecular mechanisms of 4-HBd on BBB injury induced by CIRI, focusing on its role within the RN-MMP-TJ signaling pathway. Findings from pharmacokinetic analyses and mechanistic studies collectively demonstrated that 4-HBd is a naturally derived small-molecule compound with a well-defined source, high availability, low cost, and strong brain-targeting properties. Its ability to protect the BBB appears to be mediated through anti-lipid peroxidation and oxidative stress suppression, suggesting its potential as a neuroprotective agent. Despite these promising findings, several challenges remain regarding the clinical translation of 4-HBd. The quality control of natural product-derived compounds, along with inherent limitations in their standardization and large-scale production, presents obstacles for drug development. Moreover, the metabolism and transport dynamics of 4-HBd within the brain require further investigation to optimize its therapeutic potential. The MCAO/R model employed in this study successfully replicated key pathophysiological features of IS, particularly BBB disruption. However, this model only induced MCAO, whereas human IS often involves multiple vascular territories, limiting its clinical relevance. Additionally, only male adult rats were used, which does not fully account for the complex etiology of IS in human patients, where sex, age, and comorbidities significantly influence disease progression and treatment outcomes. Future studies should expand the diversity of experimental models to better simulate the multifactorial nature of human IS. This includes incorporating sex-based differences, age-related variations, and comorbid conditions, ultimately refining preclinical drug evaluation systems to more closely align with clinical realities.

## Data Availability

The original contributions presented in the study are included in the article/Supplementary Material; further inquiries can be directed to the corresponding authors.
