# Supplementary material for: Protective effects of 4-HBd on blood–brain barrier integrity in MCAO/R model rats based on brain pharmacokinetic characteristics
Source: Front Pharmacol. 2025 Apr 8;16:1528839. doi: 10.3389/fphar.2025.1528839 (PMC12012380; doi:10.3389/fphar.2025.1528839)
Supplement: Supplementary file 1 [file Supplementaryfile1.docx]

**Behavioral Scoring**

**1. Longa 5-Point Neurological Score**

The neurological score will be assessed using the Longa modified 5-point scale within 24 hours postoperatively (See Table 1).

**Table 1: Longa 5-Point Neurological Score Sheet**

| **Longa Modified 5-Point Scale** | **scoring** | | |
| --- | --- | --- | --- |
| Without neurological deficits | | 0 | |
| Mild focal neurological deficit, characterized by the inability to extend the contralateral forepaw when the tail is lifted and the animal is suspended. | | | 1 |
| Moderate focal neurological deficit, with circling toward the contralateral side 2 | | | |
| Severe focal neurological deficit, characterized by difficulty walking and tilting toward the contralateral side. | | 3 | |
| Unable to walk independently, with decreased level of consciousness | | 4 | |
| Death | | 5 | |

**2. Neurological Damage Severity Rating Scale**

Neurological severity scoring (Neurological Severity Score, NSS) will be used for assessment at postoperative day 3 and at subsequent time points (see Table 2).

**Table 2: Neurological Severity Score (NSS) Rating Scale**

| **Motor function test** **scoring** | | | | |
| --- | --- | --- | --- | --- |
| Lift the rat by the tail (total of 3 points)  Forelimb flexion 1  Hindlimb flexion 1  Head tilted upward by more than 10° within 30 seconds 1 | | | | |
| Place the rat on the platform (Normal: 0 points; up to 3 points)  Normal crawling 0  Unable to crawl in a straight line 1  Circling toward the paretic side 2  Falling toward the paretic side 3 | | | | |
| **Sensory test scoring** | | | | |
| Superficial sensory test (visual and tactile tests) | | | 1 | |
| Proprioceptive test (deep sensation: placing the affected limb at the table edge, with no withdrawal response) | | | 1 | |
| **Horizontal bar balance test (Normal: 0 points, up to 6 points)** | | **scoring** | | |
| Balance | | | 0 | |
| Grasping the side of the horizontal bar | | | 1 | |
| Grasping the horizontal bar, with one limb falling off | | | 2 | |
| Grasping the horizontal bar, with both limbs falling off or rotating while holding the bar for >60 seconds | | | 3 | |
| Attempting to balance but failing and falling off (> 40 seconds) | | | 4 | |
| Attempting to balance but failing and falling off (< 20 seconds) | | | 5 | |
| Falling off (< 20 seconds) | | | 6 | |
| **Reflexes and abnormal movements** | **scoring** | | | |
| Pinna reflex (head shaking after stimulating the ear canal) | | | | 1 |
| Cornial reflex (blinking after cotton stimulation of the cornea) | | | | 1 |
| Startle reflex (motor reflex or screaming after hearing a sudden sound) | | | | 1 |
| Seizures, myoclonus, abnormal muscle tone | | | | 1 |
| Total | 18 | | | |

**3. Beam walking test**

The beam walking test will be used for scoring at postoperative day 3 and at subsequent time points (see Table 3).

**Table 3: Beam Walking Test Scoring Sheet**

| **Beam walking test (Normal: 0 points, up to 6 points)** | **scoring** |
| --- | --- |
| Walked successfully 0  Can walk across, occasional slipping 1  Can walk across, with more than 50% of steps slipping 2  Can walk across, but the affected hind limb does not function 3  Fell off while walking 4  Can stay on the beam but cannot walk 5  Cannot stay on the beam, falls off 6 | |

**4. Climbing rope test**

The climbing rope test will be used for scoring at postoperative day 3 and at subsequent time points (see Table 4).

**Table 4: Climbing Rope Test Scoring Sheet**

| **Climbing rope test scoring** |
| --- |
| The rat grasps a 2mm steel rope with its forelimbs for >5 seconds, with  hindlimbs also climbing the rope 0 |
| an grasp for 5 seconds, but the hindlimbs cannot climb the rope 1 |
| Can grasp for 3-4 seconds 2 |
| Can grasp for only 0-2 seconds 3 |

**5. Gait analysis test**

The gait analysis test will be used for scoring at postoperative day 3 and at subsequent time points (see Table 5).

**Table 5: Gait Analysis Test Scoring Sheet**

| **Gait analysis test (Normal: 0 points, up to 3 points) scoring** |
| --- |
| Gently lift the rat's tail tip to lift the hindlimbs, with both forelimbs  symmetrically extended, walking coordinately along a straight line 0 |
| The contralateral forelimb extends less than the ipsilateral one, 1  with slight tilting toward the opposite side while walking. |
| The contralateral forelimb shows slight extension, with the rat  circling toward the opposite side while walking. 2 |
| Unable to walk 3 |

**6. Corner test experiment**

The experimental setup consists of two 60 cm × 40 cm × 1 cm flat panels forming a 30° angle. When the experimental animal (mouse or rat) enters the deeper part of the angle, both sides of its whiskers will be stimulated, causing the rat to stand and turn to face the opening of the angle. Normal rats have an equal probability of turning left or right, while rats with unilateral brain injury primarily turn toward the side of the brain injury. Asymmetry score = (Number of 'standing turns' toward the ischemic side / Total number of 'standing turns') × 100%. The normal baseline is 50%. A higher score indicates more severe damage.
