# Supplementary material for: Protective effects of 4-HBd on blood–brain barrier integrity in MCAO/R model rats based on brain pharmacokinetic characteristics
Source: Front Pharmacol. 2025 Apr 8;16:1528839. doi: 10.3389/fphar.2025.1528839 (PMC12012380; doi:10.3389/fphar.2025.1528839)
Supplement: Supplementary file 2 [file Supplementaryfile5.docx]

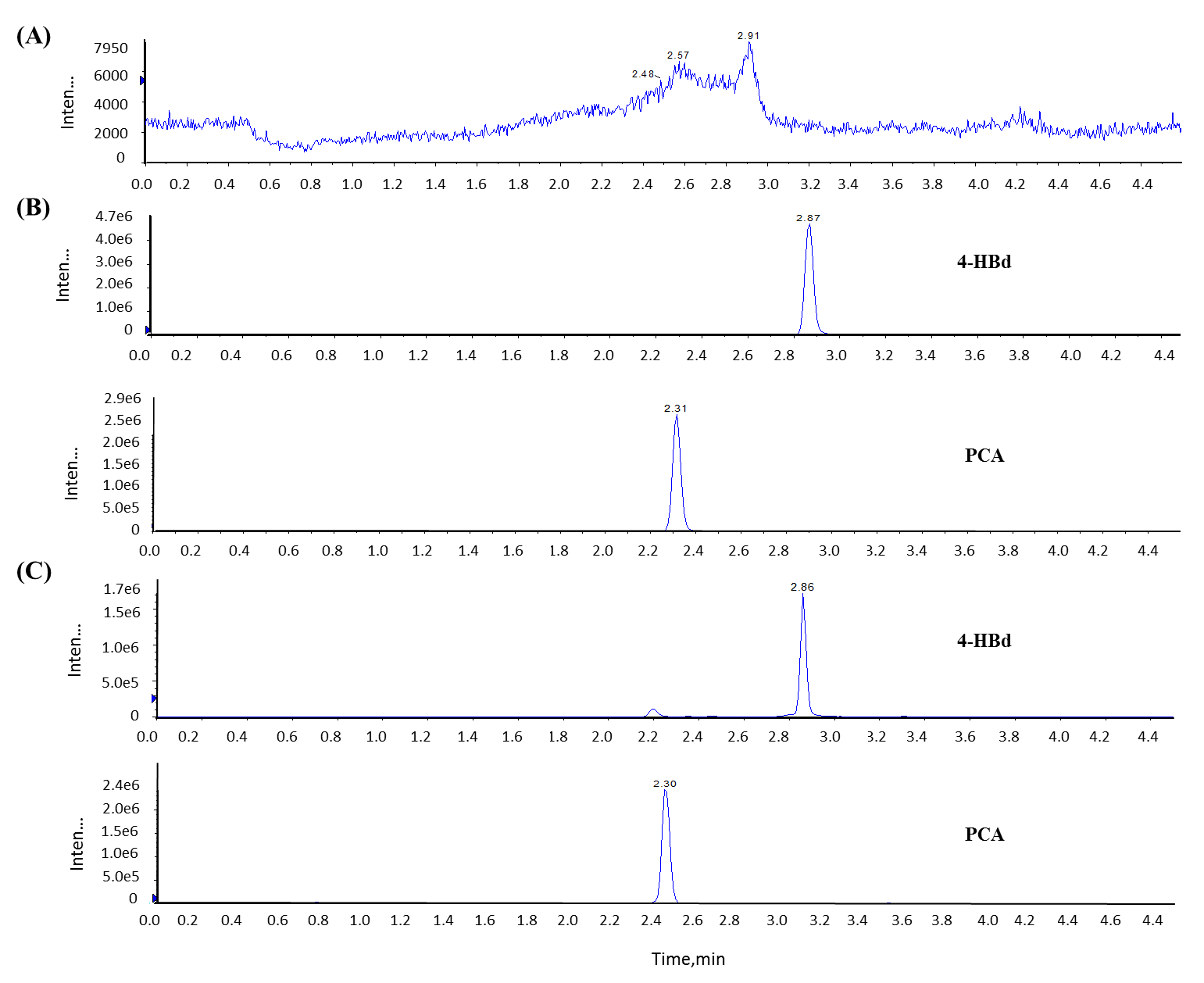


**Figure 1. Chromatogram of 4-HBd in rat brain dialysate**

**Table 1. Effect of determination method on residual rate of rat brain dialysate**

|  | 4-HBd | | PCA | |
| --- | --- | --- | --- | --- |
|  | Conc/ng·mL^-1^ | Peak area | Conc/ng·mL^-1^ | Peak area |
| ULOQ | 100 | 20900000 | 200 | 8510000 |
| DB | 0 | 71200 | 0 | 150000 |
| LLOQ | 1 | 409000 | 200 | 9650000 |
| DB/ LLOQ | 0 | 17.4% | 200 | 1.6% |

**Table 2. Lower limit of quantitation of 4-HBd in rat brain dialysate ( n = 6 )**

|  | Conc/ng·mL^-1^ | | Precision /RSD/% | | Accuracy /RE/% |
| --- | --- | --- | --- | --- | --- |
|  | Spiked | Measured (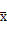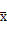±s) | Intra-day | Inter-day |  |
| 4-HBd | 1 | 0.97±0.06 | 6.73 | 6.55 | -3.1 |

**Table 3. Precision and accuracy of 4-HBd in rat brain dialysate ( n = 6 )**

|  | Conc/ng·mL^-1^ | | Precision/RSD/% | | Accuracy /RE/% |
| --- | --- | --- | --- | --- | --- |
|  | Spiked | Measured (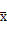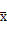±s) | Intra-day | Inter-day |  |
| 4-HBd | 2 | 1.97±0.09 | 3.94 | 8.4 | -1.3 |
|  | 20 | 20.12±0.47 | 1.61 | 5.11 | 0.6 |
|  | 80 | 73.28±1.38 | 1.46 | 3.76 | -8.4 |

**Table 4. Matrix effect of 4-HBd in rat brain dialysate ( n = 6 )**

|  | Conc/ng·mL^-1^ | Matrix effect | |  | |
| --- | --- | --- | --- | --- | --- |
|  |  | Mean | SD | RSD/% |  |
| 4-HBd | 2 | 0.43 | 0.02 | 5.47 |  |
|  | 80 | 0.61 | 0.07 | 11.23 |  |

**Table 5. Stability of 4-HBd in rat brain dialysate ( n = 3 )**

|  | Conc/ng·mL^-1^ | Room temperature stability | | Freeze**-**thaw stability | | In the Auto-sampler  for 14h stability | |
| --- | --- | --- | --- | --- | --- | --- | --- |
|  |  | Measured | RE/% | Measured | RE/% | Measured | RE/% |
| 4-HBd | 2 | 2.1 | 6.0 | 2.1 | 4.5 | 2.2 | 10.0 |
|  | 2 | 2.0 | 1.5 | 2.2 | 7.5 | 2.2 | 7.5 |
|  | 2 | 2.0 | 2.0 | 2.1 | 6.0 | 2.1 | 7.0 |
|  | 80 | 76 | -4.8 | 77 | -3.4 | 78 | -1.9 |
|  | 80 | 75 | -6.3 | 75 | -5.3 | 79 | -0.9 |
|  | 80 | 72 | -9.3 | 76 | -4.9 | 77 | -3.2 |
