# Supplementary material for: Protective effects of 4-HBd on blood–brain barrier integrity in MCAO/R model rats based on brain pharmacokinetic characteristics
Source: Front Pharmacol. 2025 Apr 8;16:1528839. doi: 10.3389/fphar.2025.1528839 (PMC12012380; doi:10.3389/fphar.2025.1528839)
Supplement: Supplementary file 3 [file Supplementaryfile3.docx]

**Table 1. Standard curve of different extraction parts of Gastrodia elata**

| test article | standard curve equation | r2 |
| --- | --- | --- |
| HBA | y= -2.58039+5206.20104X | 0.99995 |
| GAS | y= 0.54355+1385.43719X | 0.99997 |
| 4-HD | y=12.45421+83733.55170X | 0.99998 |
| 4,4-DD | y= -2.59583+5206.29878X | 0.9995 |

**Table 2. Content determination results of different extraction parts of Gastrodia elata**

| Extraction parts of Gastrodiae Rhizoma | GE2 | GE2 residue | GE2-1 | GE2-2 | GE2-3 |
| --- | --- | --- | --- | --- | --- |
| Total% | 2.134 | 0.000 | 18.350 | 0.000 | 1.219 |
