# Supplementary material for: Protective effects of 4-HBd on blood–brain barrier integrity in MCAO/R model rats based on brain pharmacokinetic characteristics
Source: Front Pharmacol. 2025 Apr 8;16:1528839. doi: 10.3389/fphar.2025.1528839 (PMC12012380; doi:10.3389/fphar.2025.1528839)
Supplement: Supplementary file 4 [file Supplementaryfile2.docx]

**1 Method validation**

**1.1 Selectivity**

Typical chromatograms of pooled blank tissues，plasma, feces, and urine were obtained from different rats. The retention time of 4-HBd in tissues was 21.5 min and in plasma, urine and faeces was 12.9 min. Results indicated that there was no apparent endogenous interference in 4-HBd determination (Figure 1-9).

**1.2 Linearity of calibration curves and LLOQ**

Calibration curves of the peak area (Y) relating to concentration (C) were prepared daily and showed good linearity in the tested ranges for 4-HBd (R2 > 0.995). The deviation of detection at LLOQ was restricted to 20% (data not shown). See Table 1 for details.

**1.3 Precision and accuracy**

Three mass control samples of 4-HBd tissue homogenate at high (781.25 μg/L), medium (195.31 μg/L) and low (6.11 μg/L) concentrations were prepared according to Section 1.7.1; three mass control samples of 4-HBd in blood, faeces and urine at high (6231.25 μg/L), medium (389.45 μg/L) and low (24.34 μg/L) concentrations were prepared. QC samples were prepared in 5 parallel for each concentration.The QC samples were tested over three successive validation batches (once a day). Five replicates for each QC level were determined in each run. The intra- and inter-day precision and accuracy results for 4-HBd are shown in Table 2, with all values within the acceptable range (deviation restricted to 10% for nine QC levels). Therefore, the determination method was reproducible and showed high accuracy, low deviation, and low relative error.

**1.4 Recovery**

The QC samples for high, medium, and low concentrations of 4-HBd were prepared. After preparation and pretreatment, 4-HBd was analyzed by HPLC, and the peak area was recorded as A1. In addition, blank plasma, fecal, and urine samples of rats were prepared. The supernatant was added to the tube containing the dried QC solution of 4-HBd. After vortex mixing, the sample was injected and determined by HPLC. The peak area of 4-HBd was recorded as A2. Recovery was calculated using A1/A2. In the matrix effect test, double-distilled water was used to replace the blank samples and the other steps were the same as the above methods. A2 was determined by HPLC, and the peak area of 4-HBd was recorded as A3. The matrix effect was calculated by A2/A3. See Table 3 for details.

**1.5 Stability**

The QC samples for high, medium, and low concentrations of 4-HBd were prepared. After pretreatment, the samples were placed at room temperature for 4 h and then at −20 °C for 7 d. The samples were frozen and thawed repeatedly (three times), then analyzed by HPLC. The relative error of accuracy and RSD were less than 15%. Results showed that 4-HBd in each sample was stable under the above three conditions. As shown in Table 4, no significant changes in the concentrations of 4-HBd were observed at the two QC levels, indicating that the analytes at the two tested concentrations exhibited acceptable stability.

**
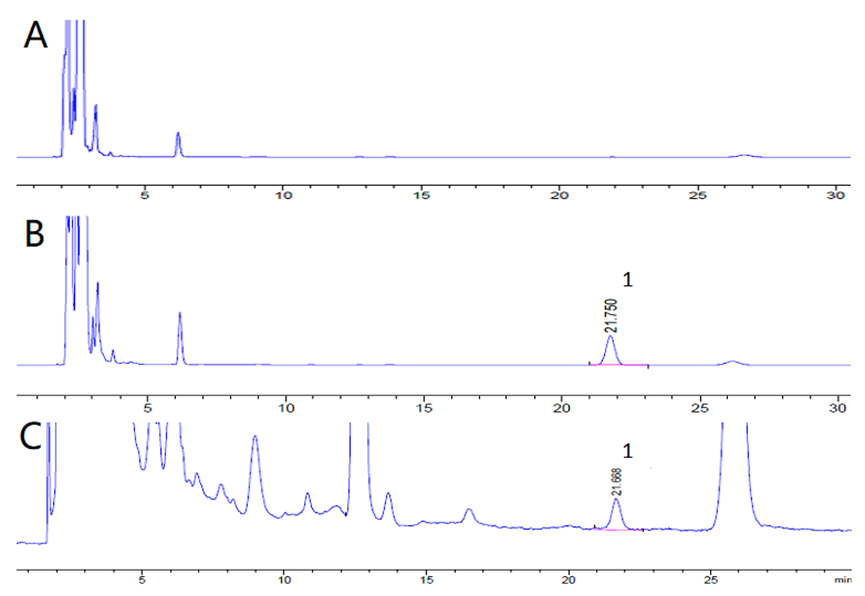
**

4-HBd

4-HBd

**Figure 1. High performance liquid chromatography of 4-HBd in heart.**

**(A. Blank heart; B. Blank heart + reference solution, 1.02 mg/mL; C. heart samples of 4-HBd by gavage in rats, ig 400mg/kg. Measured time is 30 minutes.)**

**
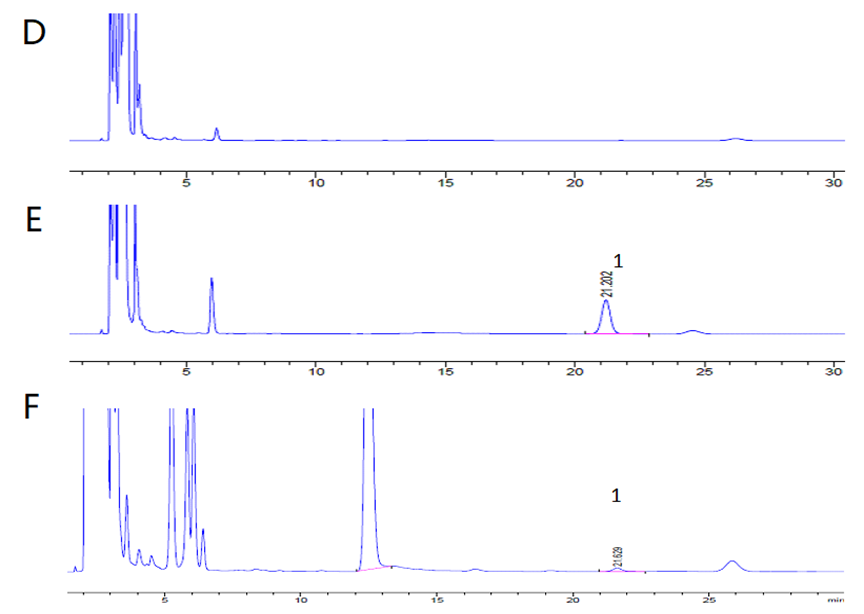
**

4-HBd

4-HBd

**Figure 2. High performance liquid chromatography of 4-HBd in liver.**

**(A. Blank liver; B. Blank liver + reference solution, 1.02 mg/mL; C. liver samples of 4-HBd by gavage in rats, ig 400mg/kg. Measured time is 30 minutes.)**

**
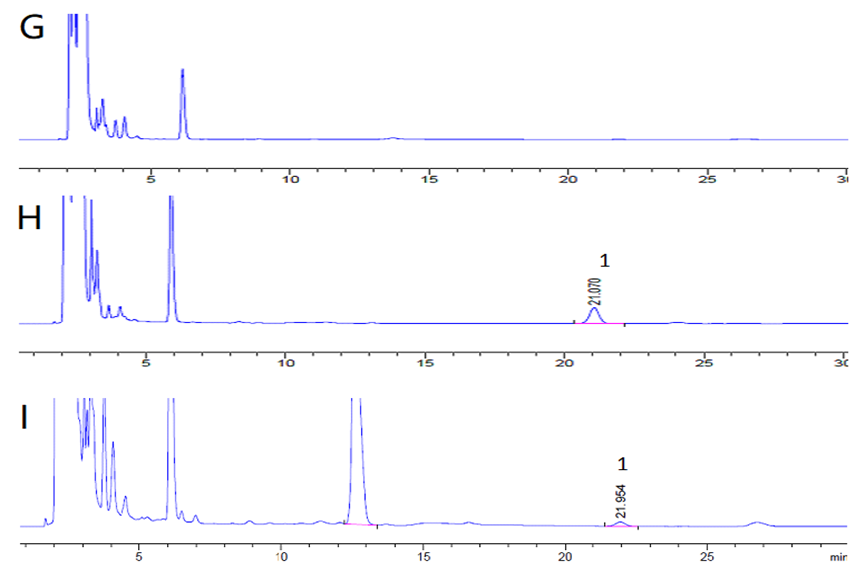
**

4-HBd

4-HBd

**Figure 3. High performance liquid chromatography of 4-HBd in spleen.**

**(A. Blank spleen; B. Blank spleen + reference solution, 1.02 mg/mL; C. spleen samples of 4-HBd by gavage in rats, ig 400mg/kg. Measured time is 30 minutes.)**

**
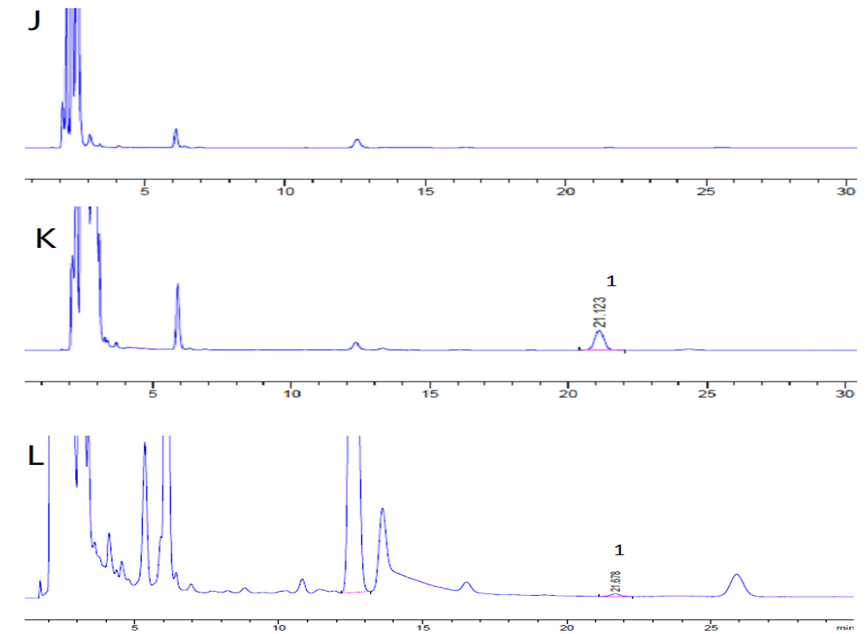
**

4-HBd

4-HBd

**Figure 4. High performance liquid chromatography of 4-HBd in lung.**

**(A. Blank lung; B. Blank lung + reference solution, 1.02 mg/mL; C. lung samples of 4-HBd by gavage in rats, ig 400mg/kg. Measured time is 30 minutes.)**

**
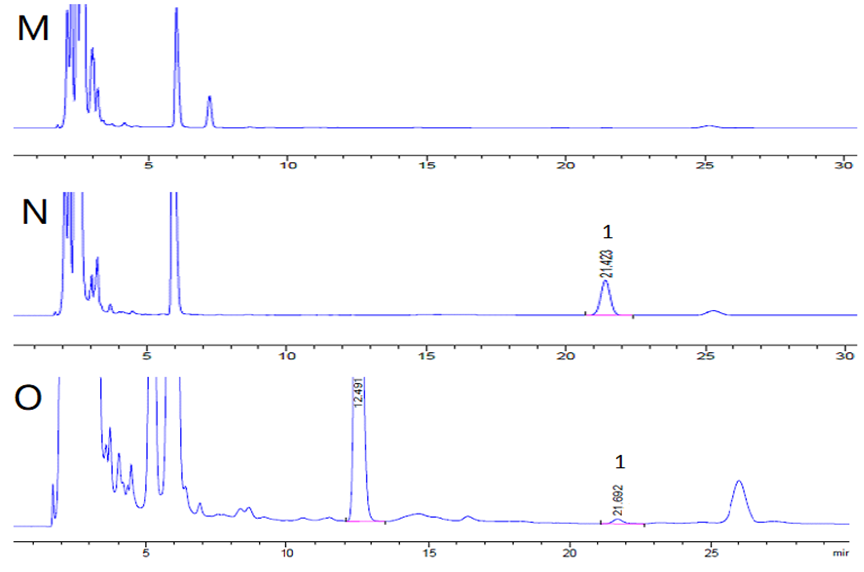
**

4-HBd

4-HBd

**Figure 5. High performance liquid chromatography of 4-HBd in kidney.**

**(A. Blank kidney; B. Blank kidney + reference solution, 1.02 mg/mL; C. kidney samples of 4-HBd by gavage in rats, ig 400mg/kg. Measured time is 30 minutes.)**

**
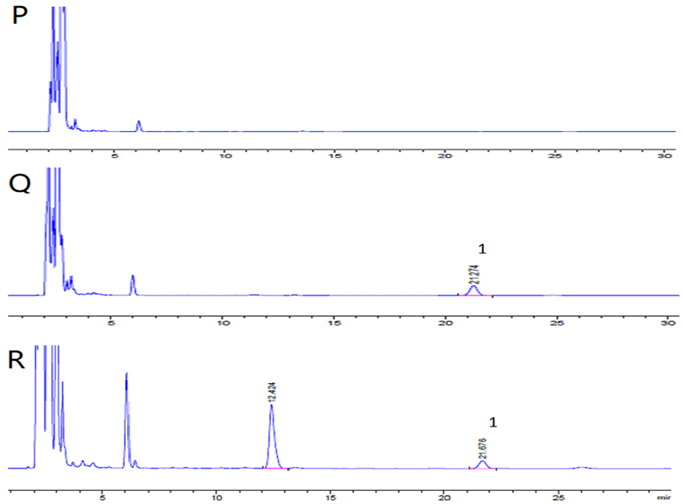
**

4-HBd

4-HBd

**Figure 6. High performance liquid chromatography of 4-HBd in brain.**

**(A. Blank brain; B. Blank brain + reference solution, 1.02 mg/mL; C. brain samples of 4-HBd by gavage in rats, ig 400mg/kg. Measured time is 30 minutes.)**

**
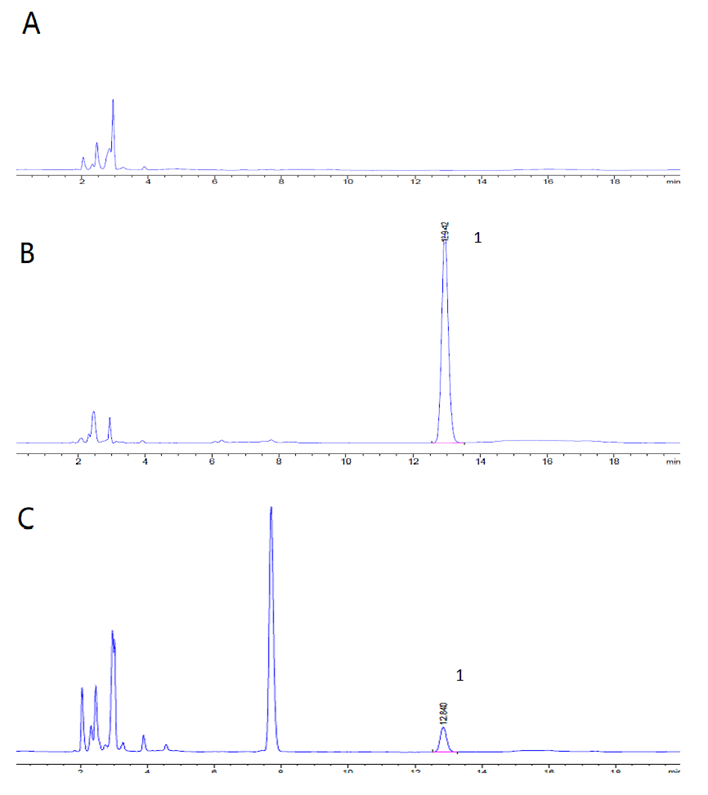
**

4-HBd

4-HBd

**Figure 7. High performance liquid chromatography of 4-HBd in plasma**

**(A. Blank plasma; B. Blank plasma + reference solution,** **1.02 mg/mL; C. Plasma samples of 4-HBd by gavage in rats, ig 400mg/kg. Measured time is 18 minutes.)**

**
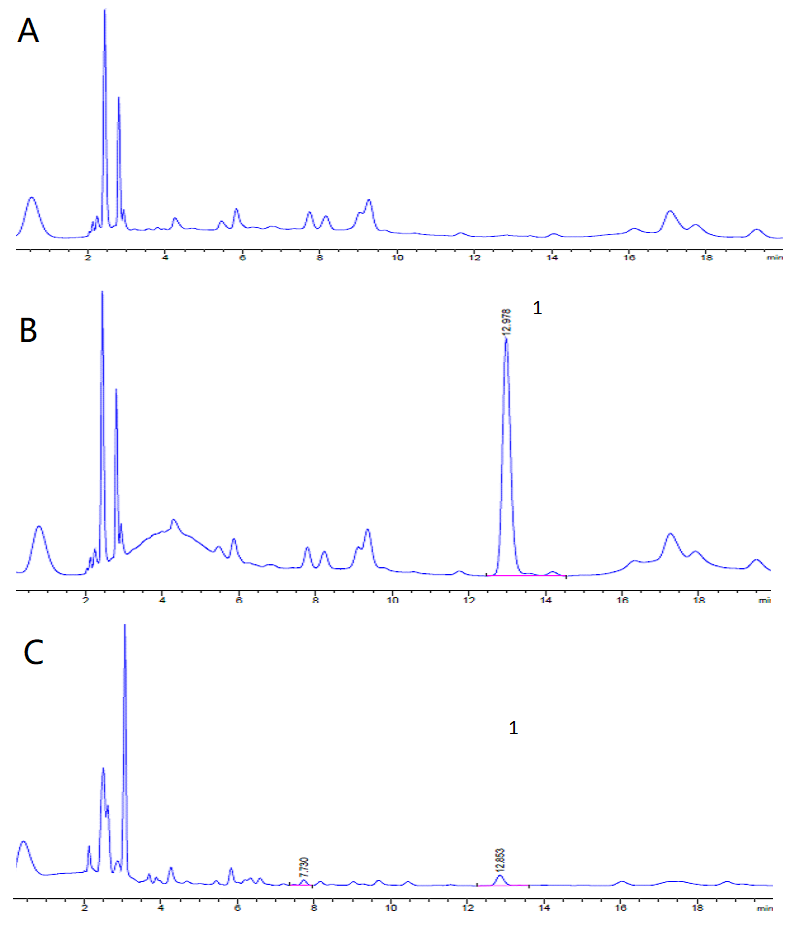
**

4-HBd

4-HBd

**Figure 8. High performance liquid chromatography of 4-HBd in feces**

**(A. Blank feces ; B. Blank feces + reference solution, 1.02mg/mL; C. Fecal samples of rats gavaged with 4-HBd,** **ig 400mg/kg. Measured time is 18 minutes.)**

**
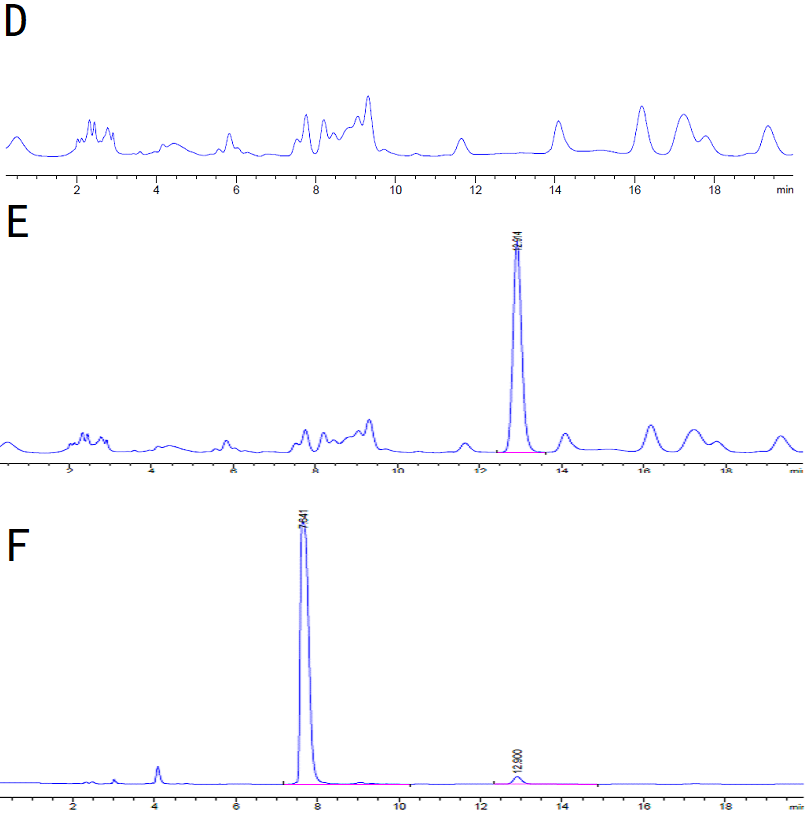
**

4-HBd

4-HBd

**Figure 9. High performance liquid chromatography of** **4-HBd in urine**

**(D. Blank urine; E.blank urine + reference solution, 1.02mg/mL; F. Urine samples of rats gavaged with 4-HBd, ig 400mg/kg. Measured time is 18 minutes.)**

**Table 1. Standard curve of 4-HBd in tissue, plasma, feces and urine**

| Sample | Standard curve | R^2^ |
| --- | --- | --- |
| heart | Y=0.35X-0.86 | 0.99 |
| liver | Y=0.42X+5.36 | 0.99 |
| spleen | Y=0.40X-0.45 | 0.99 |
| lung | Y=0.45X-1.50 | 0.99 |
| Kidney | Y=0.40X+2.64 | 0.99 |
| brain | Y=0.36X+1.00 | 0.99 |
| plasma | Y=0.21X+5.20 | 0.99 |
| feces | Y=1.37X+5.45 | 0.99 |
| urine | Y=1.27X-36.99 | 0.99 |

**Table 2. Precision and accuracy of the assay method for 4-HBd in biological samples**

| Biological sample | Conc(μg/L) | Intra-day | | | Inter-day | | | |
| --- | --- | --- | --- | --- | --- | --- | --- | --- |
|  |  | $\bar{x}$±s(μg/L) | RSD(%) | Accuracy(%) | | $\bar{x}$±s(μg/L) | (RSD/%) | Accuracy(RE%) |
| heart | 781.25 | 800.12±5.22 | 0.65 | 102.42 | | 798.02±2.00 | 0.25 | 102.15 |
|  | 195.31 | 200.83±1.47 | 0.73 | 102.83 | | 200.978±5.11 | 2.58 | 101.35 |
|  | 6.11 | 6.04±0.08 | 1.32 | 99.90 | | 6.12±0.05 | 0.79 | 100.24 |
| liver | 781.25 | 797.38±3.33 | 0.42 | 102.06 | | 796.02±1.27 | 0.16 | 101.89 |
|  | 195.31 | 201.01±2.02 | 1.00 | 102.92 | | 198.33±2.97 | 1.50 | 101.54 |
|  | 6.11 | 6.15±0.07 | 1.08 | 100.69 | | 6.08±0.07 | 1.07 | 99.62 |
| spleen | 781.25 | 784.93±13.35 | 1.70 | 100.47 | | 785.62±1.96 | 0.25 | 100.56 |
|  | 195.31 | 189.53±5.36 | 2.83 | 97.04 | | 191.47±1.74 | 0.91 | 98.03 |
|  | 6.11 | 6.01±0.24 | 3.94 | 98.33 | | 6.12±0.10 | 1.66 | 100.20 |
| lung | 781.25 | 790.63±3.80 | 0.48 | 101.20 | | 790.53±0.69 | 0.09 | 101.19 |
|  | 195.31 | 198.89±1.09 | 0.55 | 101.83 | | 198.92±1.15 | 0.58 | 101.85 |
|  | 6.11 | 6.12±0.04 | 0.63 | 100.23 | | 6.126±0.06 | 0.90 | 100.26 |
| kidney | 781.25 | 794.42±3.74 | 0.47 | 101.69 | | 796.18±1.54 | 0.19 | 101.91 |
|  | 195.31 | 199.60±1.92 | 0.96 | 102.20 | | 199.02±0.63 | 0.32 | 101.90 |
|  | 6.11 | 6.16±0.08 | 1.34 | 100.79 | | 6.13±0.13 | 2.15 | 100.29 |
| brain | 781.25 | 793.10±1.46 | 0.18 | 101.52 | | 793.57±1.45 | 0.18 | 101.58 |
|  | 195.31 | 197.49±0.99 | 0.50 | 101.12 | | 198.10±0.74 | 0.37 | 101.43 |
|  | 6.11 | 6.11±0.1 | 1.62 | 100.03 | | 6.11±0.05 | 0.85 | 99.99 |
| plasma | 6231.25 | 6149.35±60.03 | 0.98 | 98.69 | | 6140.66±26.10 | 0.43 | 97.17 |
|  | 389.45 | 380.12±4.97 | 1.31 | 97.60 | | 379.81±0.27 | 0.07 | 96.49 |
|  | 24.34 | 23.41±1.27 | 5.41 | 96.18 | | 23.29±0.11 | 0.05 | 94.83 |
| feces | 6231.25 | 6067.62±44.97 | 0.74 | 97.37 | | 6049.57±17.65 | 0.29 | 97.08 |
|  | 389.45 | 375.27±4.76 | 1.27 | 96.36 | | 374.43±1.24 | 0.33 | 96.14 |
|  | 24.34 | 23.16±0.45 | 1.93 | 95.15 | | 23.09±0.07 | 0.29 | 94.85 |
| urine | 6231.25 | 6053.09±43.86 | 0.72 | 97.14 | | 6055.17±3.39 | 0.06 | 97.17 |
|  | 389.45 | 377.33±5.10 | 1.35 | 96.89 | | 375.78±1.35 | 0.36 | 96.49 |
|  | 24.34 | 23.17±0.65 | 2.78 | 95.20 | | 23.08±0.08 | 0.35 | 94.83 |

**Table 3. Recovery and matrix effects of 4-HBd in tissue, plasma, feces and urine**

| Biological sample | Conc(μg/L) | Recovery(%) | | Matric effect(%) | |
| --- | --- | --- | --- | --- | --- |
|  |  | ±s(%) 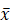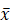 | RSD/% | ±s(%) 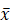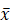 | RSD/% |
| heart | 781.25 | 96.02±0.16 | 0.17 | 99.72±0.35 | 0.35 |
|  | 195.31 | 92.94±1.14 | 1.23 | 99.42±1.71 | 1.72 |
|  | 6.11 | 91.02±0.64 | 0.71 | 1.00±1.44 | 1.43 |
| liver | 781.25 | 95.67±0.16 | 0.16 | 100.20±0.34 | 0.34 |
|  | 195.31 | 94.98±2.34 | 2.46 | 100.13±1.44 | 1.44 |
|  | 6.11 | 91.11±2.84 | 3.12 | 99.21±2.90 | 2.92 |
| spleen | 781.25 | 95.89±0.35 | 0.37 | 100.14±0.36 | 0.35 |
|  | 195.31 | 94.62±0.95 | 0.10 | 100.74±1.57 | 1.56 |
|  | 6.11 | 92.20±3.55 | 3.85 | 99.56±1.34 | 1.34 |
| lung | 781.25 | 96.23±0.22 | 0.23 | 100.09±0.34 | 0.34 |
|  | 195.31 | 95.49±1.36 | 1.42 | 100.70±1.15 | 1.15 |
|  | 6.11 | 91.37±3.33 | 3.64 | 99.60±2.80 | 2.81 |
| kidney | 781.25 | 96.17±0.22 | 0.22 | 100.20±0.28 | 0.28 |
|  | 195.31 | 95.05±0.70 | 0.74 | 101.30±1.45 | 1.43 |
|  | 6.11 | 90.64±2.15 | 2.37 | 97.87±3.01 | 3.07 |
| brain | 781.25 | 96.17±0.22 | 0.22 | 100.20±0.28 | 0.28 |
|  | 195.31 | 95.05±0.70 | 0.74 | 101.30±1.45 | 1.43 |
|  | 6.11 | 90.64±2.15 | 2.37 | 97.87±3.01 | 3.07 |
| plasma | 6231.25 | 87.55±0.83 | 0.94 | 99.57±1.01 | 1.02 |
|  | 389.45 | 88.12±4.99 | 5.67 | 97.94±2.80 | 2.86 |
|  | 24.34 | 93.97±5.07 | 5.40 | 1.01±7.56 | 7.50 |
| feces | 6231.25 | 93.23±0.61 | 93.23 | 99.77±0.86 | 99.77 |
|  | 389.45 | 94.91±0.69 | 94.91 | 100.04±0.19 | 100.04 |
|  | 24.34 | 93.85±2.05 | 93.85 | 99.54±2.61 | 99.54 |
| urine | 6231.25 | 92.76±0.42 | 92.76 | 100.16±0.48 | 100.16 |
|  | 389.45 | 95.59±0.81 | 95.59 | 99.99±0.21 | 99.99 |
|  | 24.34 | 95.31±2.75 | 95.31 | 99.07±0.96 | 99.07 |

**Table 4. Stability of 4-HBd in rat plasma , feces and urine under different storage conditions**

| Biological sample | Conc  (μg/L) | Room temperature for 4h | | | | -20 °C for 7 days | | | Three freeze–thaw cycles | | | | |
| --- | --- | --- | --- | --- | --- | --- | --- | --- | --- | --- | --- | --- | --- |
|  |  | ±s 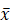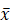 (μg/L) | Accuracy  (RE%) | RSD/% | ±s 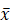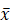 (μg/L) | | Accuracy  (RE%) | RSD/  % | | ±s 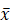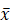 (μg/L) | Accuracy  (RE%) | RSD/  % |  |
| heart | 781.25 | 801.49±2.09 | 102.59 | 0.26 | 801.52±2.05 | | 102.59 | 0.26 | | 802.09±1.20 | 102.67 | 0.15 |  |
|  | 195.31 | 201.26±2.03 | 103.05 | 1.01 | 201.83±1.51 | | 103.33 | 0.75 | | 202.44±1.71 | 103.65 | 0.84 |  |
|  | 6.11 | 6.12±0.7 | 100.10 | 1.11 | 6.12±0.10 | | 100.20 | 1.59 | | 6.21±0.04 | 101.57 | 0.61 |  |
| liver | 781.25 | 798.70±1.55 | 102.23 | 0.19 | 798.93±1.77 | | 102.26 | 0.22 | | 799.72±1.25 | 102.36 | 0.16 |  |
|  | 195.31 | 198.98±1.78 | 101.88 | 0.90 | 199.07±1.47 | | 101.92 | 0.74 | | 200.17±.21 | 102.50 | 0.60 |  |
|  | 6.11 | 6.12±0.03 | 100.23 | 0.52 | 6.15±0.04 | | 100.60 | 0.67 | | 6.14±0.04 | 100.52 | 0.71 |  |
| spleen | 781.25 | 795.35±2.72 | 101.81 | 0.34 | 796.84±2.42 | | 102.00 | 0.30 | | 796.41±3.06 | 101.94 | 0.38 |  |
|  | 195.31 | 196.08±2.78 | 100.40 | 1.42 | 196.21±2.84 | | 100.46 | 1.45 | | 197.20±1.93 | 100.97 | 0.98 |  |
|  | 6.11 | 6.02±0.60 | 98.56 | 0.99 | 6.02±0.04 | | 98.49 | 0.72 | | 6.06±0.06 | 99.12 | 1.07 |  |
| lung | 781.25 | 794.80±1.72 | 101.73 | 0.22 | 794.906±1.34 | | 101.75 | 0.17 | | 795.53±1.93 | 101.83 | 0.24 |  |
|  | 195.31 | 197.46±0.90 | 101.10 | 0.45 | 196.46±1.39 | | 100.59 | 0.71 | | 197.39±0.76 | 101.07 | 0.38 |  |
|  | 6.11 | 6.11±0.10 | 99.93 | 1.56 | 6.14±0.05 | | 100.46 | 0.74 | | 4.15±0.08 | 100.59 | 1.25 |  |
| kidney | 781.25 | 795.89±1.39 | 101.87 | 0.17 | 796.61±1.56 | | 101.97 | 0.20 | | 796.97±4.44 | 102.01 | 0.18 |  |
|  | 195.31 | 199.32±0.86 | 102.05 | 0.43 | 197.78±0.99 | | 101.26 | 0.50 | | 199.50±1.15 | 102.14 | 0.58 |  |
|  | 6.11 | 6.12±0.06 | 100.13 | 0.95 | 6.13±0.05 | | 100.26 | 0.84 | | 6.13±0.07 | 100.29 | 1.19 |  |
| brain | 781.25 | 792.71±0.98 | 101.47 | 0.12 | 794.78±1.02 | | 101.73 | 0.13 | | 795.40±0.76 | 101.81 | 0.10 |  |
|  | 195.31 | 197.54±0.91 | 101.14 | 0.46 | 197.53±0.76 | | 101.14 | 0.39 | | 198.14±0.51 | 101.45 | 0.26 |  |
|  | 6.11 | 6.05±0.16 | 99.08 | 2.69 | 6.09±0.04 | | 99.71 | 1.17 | | 6.14±0.08 | 100.46 | 1.32 |  |
| plasma | 6231.25 | 6112.06±48.53 | 98.09 | 0.79 | 6145.02±75.05 | | 98.62 | 1.22 | | 6140.86±82.12 | 98.55 | 1.34 |  |
|  | 389.45 | 382.23±3.59 | 98.15 | 0.94 | 379.80±4.03 | | 97.52 | 1.06 | | 380.57±3.45 | 97.72 | 0.91 |  |
|  | 24.34 | 23.16±0.51 | 95.14 | 2.22 | 23.25±0.68 | | 95.51 | 2.92 | | 23.12±0.59 | 94.98 | 2.56 |  |
| feces | 6231.25 | 6073.52±56.15 | 97.47 | 0.92 | 6072.67±50.62 | | 97.45 | 0.83 | | 6079.08±85.90 | 97.56 | 1.41 |  |
|  | 389.45 | 376.11±3.95 | 96.57 | 1.05 | 377.49±5.16 | | 96.93 | 1.37 | | 377.11±6.38 | 96.83 | 1.69 |  |
|  | 24.34 | 23.24±0.62 | 95.49 | 2.67 | 23.16±0.53 | | 95.15 | 2.30 | | 23.13±0.68 | 95.02 | 2.92 |  |
| urine | 6231.25 | 6087.33±73.58 | 97.69 | 1.21 | 6082.14±47.65 | | 97.61 | 0.78 | | 6087.07±59.41 | 97.69 | 0.98 |  |
|  | 389.45 | 376.90±5.41 | 96.78 | 1.44 | 376.07±7.51 | | 96.56 | 2.00 | | 376.58±4.67 | 96.78 | 1.24 |  |
|  | 24.34 | 23.28±0.47 | 95.65 | 2.03 | 23.42±0.57 | | 96.22 | 2.44 | | 22.68±23.24 | 95.47 | 2.16 |  |
