# Supplementary material for: Protective effects of 4-HBd on blood–brain barrier integrity in MCAO/R model rats based on brain pharmacokinetic characteristics
Source: Front Pharmacol. 2025 Apr 8;16:1528839. doi: 10.3389/fphar.2025.1528839 (PMC12012380; doi:10.3389/fphar.2025.1528839)
Supplement: Supplementary file 5 [file Supplementaryfile4.docx]

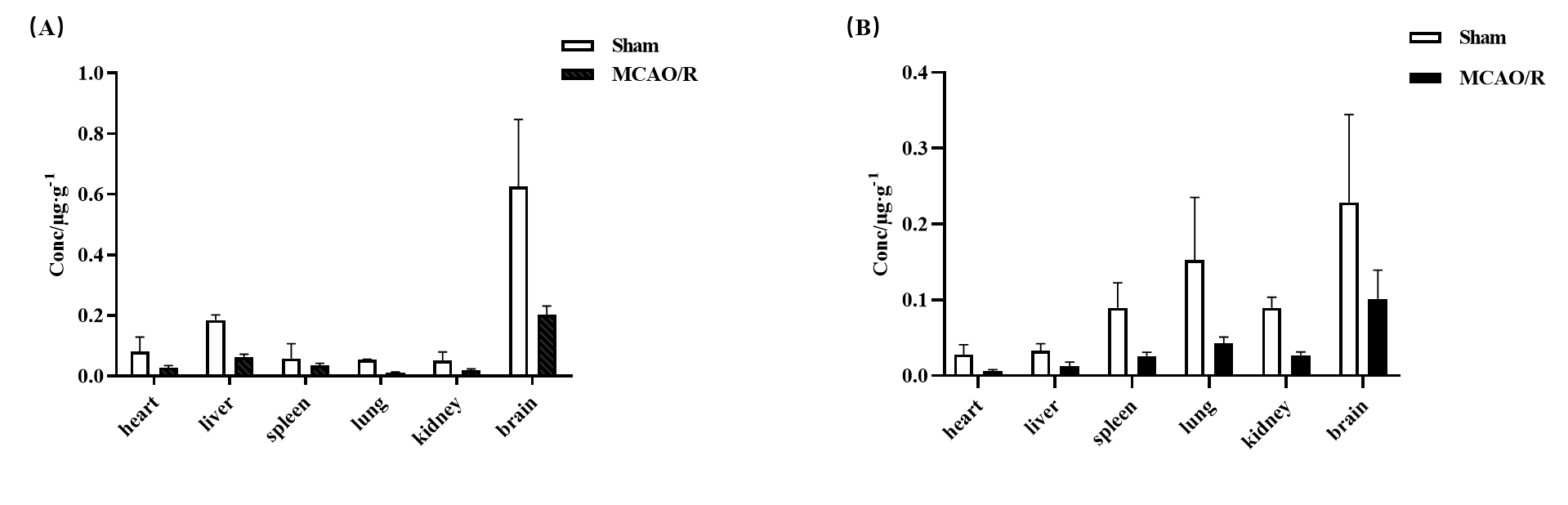
**Figure (A). Distribution of the equilibrium phase (4 min) of ig. 4-HBd in rats Figure (B). Distribution of the elimination phase (1h) of ig. 4-HBd in rats**
